# Supplementary material for: Solvent-Dependent Structures of Natural Products Based on the Combined Use of DFT Calculations and 1H-NMR Chemical Shifts
Source: Molecules. 2019 Jun 20;24(12):2290. doi: 10.3390/molecules24122290 (PMC6631582; doi:10.3390/molecules24122290)
Supplement: Supplementary file 1 [file molecules-24-02290-s001.pdf]

Solvent-dependent structures of natural products based on the combined use of DFT calculations and ^{1}H-NMR chemical shifts

Saima H. Mari[1], Panayiotis C. Varras[2], Atia-tul-Wahab*[3], Iqbal M. Choudhary[1,3], Michael G. Siskos[2], Ioannis P. Gerothanassis*[1,2,3]

[1] H.E.J. Research Institute of chemistry, International Center for Chemical and Biological Sciences, University of Karachi, Karachi 7527, Pakistan

[2] Section of Organic Chemistry & Biochemistry, Department of Chemistry, University of Ioannina, Ioannina, GR-45110, Greece

[3] Dr. Panjwani Center for Molecular Medicine and Drug Research, International Center for Chemical and Biological Sciences, University of Karachi, Karachi 7527, Pakistan

* Corresponding authors: Atia-tul-Wahab, e-mail: Atiatulwahab@gmail.com; I.P. Gerothanassis, email: igeroth@uoi.gr

Supplementary Material

Table S1. ^{13}C-NMR chemical shifts of emodin (**2**) in $CDCl_3$, acetone-d_6 and DMSO-d_6.

Carbon atoms	$CDCl_3$	Acetone-d_6	DMSO-d_6
C-1	164.8	165.8	164.5
C-8	162.5	162.0	161.5
C-2	109.6	108.2	108.0
C-3	165.8	165.6	165.6
C-4	110.5	108.8	108.8
C-5	122.1	120.0	120.6
C-6	148.7	148.1	148.4
C-7	126.0	124.2	124.2
C-9	188.9	189.0	189.8
C-10	182.9	181.1	181.5
C-11	110.1	109.1	109.1
C-12	135.0	135.1	133.2
C-13	113.8	113.0	113.5
C-14	132.9	132.0	132.9

Table S2. Calculated ^{1}H-NMR chemic/al shifts, δ_{calc}, of chrysophanol (**1**), emodin (**2**), and physcion (**3**) using the CPCM in $CHCl_3$, acetone and DMSO.

Compound	Solvent	Group	B3LYp 6-31+G(d)	ωB97XD/ 6-31+G(d)	APFD/ 6-31+G(d)	M062X/ Def2TZVP	TPSSh/ TZVP
**Chrysophanol (1)**	$CHCl_3$	C(1)-OH	12.37	11.85	12.63	11.94	13.15
		C(8)-OH	12.40	12.04	12.65	11.95	13.13
		C(4)-H	8.05	8.11	8.09	8.12	8.07
		C(5)-H	8.17	8.17	8.21	8.24	8.19
		C(3)-H	7.90	7.90	7.91	7.94	7.90
		C(7)-H	7.39	7.37	7.40	7.43	7.40
		C(2)-H	7.54	7.54	7.56	7.58	7.55
		C(6)-CH_3	2.49	2.48	2.53	2.55	2.49
**Emodin (2)**		C(1)-OH	12.43	11.86	12.53	11.91	13.44
**isomer A**		C(8)-OH	12.32	11.82	12.56	11.86	13.42
		C(5)-H	8.08	8.07	7.52	7.53	7.55
		C(4)-H	7.64	7.47	7.42	7.46	7.57
		C(7)-H	7.41	7.41	8.09	8.14	8.22
		C(2)-H	6.74	6.95	7.04	7.02	7.21
		C(3)-OH	5.24	5.09	5.17	5.16	5.59
		C(6)-CH_3	2.53	2.49	2.55	2.57	2.69
**Emodin (2)**		C(1)-OH	12.28	11.98	12.69	12.06	13.26

**isomer B**		C(8)-OH	12.31	11.84	12.57	11.87	13.09
		C(5)-H	8.07	8.09	8.11	7.69	8.10
		C(4)-H	7.49	7.63	7.68	7.46	7.66
		C(7)-H	7.41	7.41	7.42	8.15	7.42
		C(2)-H	7.00	6.70	6.73	6.75	6.74
		C(3)-OH	5.22	5.12	5.20	5.16	5.20
		C(6)-CH_3	2.52	2.41	2.55	2.57	2.52
**Physcion (3)**		C(1)-OH	12.23	11.83	12.47	11.86	12.98
**isomerA**		C(8)-OH	12.34	11.87	12.60	11.90	13.13
		C(2)-H	6.94	6.91	6.95	6.96	6.94
		C(4)-H	7.73	7.75	7.79	7.83	7.79
		C(5)-H	8.09	8.09	8.12	8.15	8.11
		C(7)-H	7.37	7.37	7.38	7.41	7.38
		C(3)-OCH	4.00	4.05	4.01	4.06	4.02
		C(6)-CH_3	2.47	2.45	2.50	2.51	2.47
**Physcion (3)**		C(1)-OH	12.69	12.21	12.95	12.33	13.50
isomerB		C(8)-OH	12.27	11.80	12.54	11.85	13.05
		C(2)-H	6.88	6.87	6.90	6.96	6.91
		C(4)-H	7.62	7.61	7.66	7.67	7.63
		C(5)-H	8.09	8.09	8.12	8.15	8.10
		C(7)-H	7.40	7.38	7.41	7.44	7.40
		C(3)-OCH	3.99	3.97	3.99	4.03	4.00
		C(6)-CH_3	2.52	2.52	2.55	2.57	2.52
Chrysophanol **(1)**	Acetone	C(1)-OH	12.37	11.96	12.63	11.94	13.15
		C(8)-OH	12.40	11.92	12.65	11.95	13.13
		C(4)-H	8.05	8.05	8.09	8.12	8.08
		C(5)-H	8.18	8.18	8.22	8.24	8.20
		C(3)-H	7.93	7.91	7.96	7.98	7.96
		C(7)-H	7.41	7.41	7.43	7.46	7.44
		C(2)-H	7.57	7.55	7.59	7.61	7.59
		C(6)-CH_3	2.52	2.54	2.55	2.56	2.52
Emodin **(2)**		C(1)-OH	12.27	11.85	12.52	11.90	13.05
Isomer A		C(8)-OH	12.28	11.81	12.55	11.85	13.06
		C(5)-H	8.07	8.08	8.11	8.13	8.10
		C(4)-H	7.52	7.52	7.57	7.56	7.56
		C(7)-H	7.43	7.44	7.45	7.48	7.45

		C(2)-H	7.00	6.96	7.01	7.02	7.01
		C(3)-OH	5.38	5.25	5.33	5.30	5.34
		C(6)-CH3	2.53	2.51	2.57	2.58	2.58
Emodin **(2)**		C(1)-OH	12.41	11.96	12.67	12.05	13.25
Isomer B		C(8)-OH	12.31	11.83	12.57	11.86	13.08
		C(5)-H	8.08	8.09	8.12	8.15	8.11
		C(4)-H	7.62	7.61	7.66	7.67	7.66
		C(7)-H	7.43	7.44	7.45	7.48	7.46
		C(2)-H	6.81	6.78	6.81	6.82	6.82
		C(3)-OH	5.41	5.28	5.37	5.33	5.38
		C(6)-CH3	2.54	2.52	2.57	2.59	2.55
Physcion **(3)**		C(1)-OH	12.22	11.90	12.54	11.85	12.95
Isomer A		C(8)-OH	12.32	11.93	12.66	11.88	13.09
		C(2)-H	6.93	6.99	7.03	6.97	6.93
		C(4)-H	7.75	7.85	7.89	7.85	7.80
		C(5)-H	8.09	8.17	8.21	8.16	8.10
		C(7)-H	7.39	7.47	7.49	7.44	7.39
		C(3)-OCH	4.02	4.09	4.1	4.08	4.03
		C(6)-CH3	2.48	2.54	2.60	2.53	2.47
Physcion **(3)**		C(1)-OH	12.67	12.20	13.01	12.31	13.47
Isomer B		C(8)-OH	12.26	11.80	12.60	11.85	13.03
		C(2)-H	6.93	6.94	7.03	7.01	6.96
		C(4)-H	7.60	7.59	7.72	7.65	7.61
		C(5)-H	8.09	8.10	8.20	8.16	8.10
		C(7)-H	7.42	7.41	7.52	7.47	7.42
		C(3)-OCH	4.01	4.00	4.09	4.05	4.01
		C(6)-CH3	2.53	2.54	2.64	2.58	2.52
Chrysophanol **(1)**	DMSO	C(1)-OH	12.36	11.96	12.62	11.94	13.15
		C(8)-OH	12.35	11.92	12.64	11.95	13.13
		C(4)-H	8.16	8.05	8.08	8.12	8.08
		C(5)-H	8.03	8.18	8.21	8.24	8.20
		C(3)-H	7.92	7.92	7.95	7.99	7.97
		C(7)-H	7.40	7.41	7.43	7.47	7.45
		C(2)-H	7.56	7.56	7.59	7.61	7.60

		C(6)-CH_3	2.50	2.55	2.54	2.57	2.53
Emodin **(2)**		C(1)-OH	12.26	11.85	12.52	11.90	13.06
Isomer A		C(8)-OH	12.28	11.81	12.54	11.85	13.07
		C(5)-H	8.07	8.08	8.11	8.14	8.10
		C(4)-H	7.53	7.53	7.57	7.57	7.57
		C(7)-H	7.44	7.45	7.45	7.49	7.46
		C(2)-H	7.00	6.97	7.00	7.02	7.01
		C(3)-OH	5.41	5.29	5.36	5.33	5.37
		C(6)-CH_3	2.54	2.51	2.57	2.59	2.54
Emodin **(2)**		C(1)-OH	12.41	11.97	12.61	12.05	13.25
Isomer B		C(8)-OH	12.30	11.83	12.55	11.86	13.08
		C(5)-H	8.08	8.10	8.11	8.15	8.11
		C(4)-H	7.62	7.61	7.65	7.66	7.66
		C(7)-H	7.44	7.45	7.45	7.49	7.47
		C(2)-H	6.82	6.79	6.81	6.84	6.83
		C(3)-OH	5.44	5.31	5.39	5.36	5.41
		C(6)-CH_3	2.54	2.52	2.56	2.59	2.55
Physcion **(3)**		C(1)-OH	12.37	11.82	12.46	11.85	12.97
Isomer A		C(8)-OH	12.45	11.85	12.58	11.88	13.11
		C(2)-H	6.93	6.91	6.95	6.97	6.95
		C(4)-H	7.62	7.78	7.82	7.86	7.83
		C(5)-H	7.88	8.10	8.13	8.16	8.13
		C(7)-H	7.29	7.40	7.42	7.44	7.42
		C(3)-OCH_3	4.07	4.02	4.03	4.08	4.06
		C(6)-CH_3	2.47	2.47	2.52	2.54	2.50
Physcion **(3)**		C(1)-OH	12.67	12.20	12.87	12.32	13.50
Isomer B		C(8)-OH	12.25	11.80	12.51	11,85	13.05
		C(2)-H	6.94	6.94	6.96	7.02	6.99
		C(4)-H	7.59	7.59	7.63	7.65	7.63
		C(5)-H	8.09	8.10	8.12	8.16	8.12
		C(7)-H	7.43	7.42	7.44	7.48	7.45
		C(3)-OCH_3	4.02	4.01	4.01	4.06	4.04
		C(6)-CH_3	2.53	2.54	2.56	2.59	2.55

Table S3. Electronic energy (Hartree units) and ΔG_A-ΔG_B values of conformers A and B of emodin (**2**) (Figure 4) and conformers A and B of physcion (**3**) (Figure 8) using various functionals and basis sets (gas phase).

Compound	Method	Conformer A	Conformer B	ΔG_A-ΔG_B (Kcal/mol)
**Emodin (2)**	B3LYP/6-31+G(d)	-953.636853	-953.636469	-0.24
	ωB97XD/6-31+G(d)	-953.316865	-953.316600	-0.166
	APFD/6-31+G(d)	-952.887571	-952.887259	-0.196
	M06-2X/Def2TZVP	-953.607879	-953.607585	-0.184
	TPSSh/TZVP	-953.971358	-953.971183	-0.110
**Physcion (3)**	B3LYP/6-31+G(d)	-992.915578	-992.915690	0.070
	ωB97XD/6-31+G(d)	-992.582078	-992.581941	0.086
	APFD/6-31+G(d)	-992.131829	-992.132167	0.212
	M06-2X/Def2TZVP	-992.874683	-992.875785	0.692
	TPSSh/TZVP	-993.262823	-993.263309	0.305

Table S4. Dipole moments (μ) in Debye, energies including zero-point energy (E_0) in Hartree units, and energy differences ($\Delta E_0 = E_A$-E_B in kcal/mol) for optimized structures of conformers A and B of emodin (**2**) with one discrete solvent molecule in the corresponding solvent using the continuum model, PCPM and the B3LYP functional along with the 6-31+G(d) basis set.

Solvation complex	μ/D	E_0/Hartrees	$\Delta E_0 = E_A$-E_B (kcal/mol)	ΔG (kcal/mol)
Emodin+$CHCl_3$/chloroform solvent, Conf A	4.87	-2372.87310	-0.04	+0.17
Emodin+$CHCl_3$/chloroform solvent, Conf B	1.80	-2372.87303		
Emodin+$(CH_3)_2CO$/acetone solvent, Conf. A	8.58	-1146.70587	-0.13	-0.126
Emodin+$(CH_3)_2CO$/acetone solvent, Conf. B	6.71	-1146.70607		
Emodin+DMSO/DMSO solvent, Conf. A_1	10.00	-1506.75472	0.13	-0.32
Emodin+DMSO/DMSO solvent, Conf. B	9.26	-1506.75492		

Table S5. Calculated ^{1}H-NMR chemical shifts, δ_{calc}, and δ_{exp} - δ_{calc} of emodin (**2**) with the inclusion of single discrete solvent molecule at the C(3)–OH group.

Conformer A	Groups	$\delta_{calc, CHCl_3}$	$\delta_{exp, CHCl_3}$ - $\delta_{Calc, CHCl_3}$	$\delta_{calc, acetone}$	$\delta_{exp, acetone}$ - $\delta_{Calc, acetone}$	$\delta_{calc, DMSO}$[a]	$\delta_{exp, DMSO}$ - $\delta_{Calc, DMSO}$
	C(1)-OH	12.56	-0.30	12.59	-0.38	12.55	-0.44
	C(8)-OH	12.35	-0.27	12.41	-0.32	12.53	-0.49
	C(5)-H	7.98	-0.37	7.95	-0.37	7.95	-0.45

	C(4)-H	7.49	-0.23	7.53	-0.26	7.71	-0.53
	C(7)-H	7.35	-0.28	7.41	-0.26	7.38	-0.27
	C(2)-H	7.10	-0.45	6.89	-0.22	6.80	-0.23
	C(3)-OH	5.42	0.76	11.25	-1.04	11.68	-0.27
	C(6)-CH_3	2.47	-0.06	2.45	0.02	2.47	-0.05
**Conformer B**							
	C(1)-OH	12.55	-0.29	12.57	-0.36	12.60	-0.49
	C(8)-OH	12.50	-0.42	12.47	-0.38	12.41	-0.37
	C(5)-H	8.00	-0.39	8.02	-0.44	8.03	-0.53
	C(4)-H	7.73	-0.47	7.58	-0.31	7.52	-0.34
	C(7)-H	7.37	-0.30	7.42	-0.27	7.41	-0.30
	C(2)-H	6.75	-0.10	6.93	-0.26	7.02	-0.45
	C(3)-OH	5.45	0.73	11.26	-1.05	11.71	-0.30
	C(6)-CH_3	2.48	-0.07	2.53	-0.06	2.52	-0.11

[a] conformer A_1

Table S6. Comparison of the structural data of minimized structures of emodin (**2**) using the B3LYP functional with 6-31+G(d) basis set in three solvents $CHCl_3$, acetone, and DMSO with the structural data of single-crystal X-ray structure [55].

Bond length	B3LYP/6-31+G(d)	B3LYP/6-31+G(d) + DMSO	B3LYP/6-31+G(d) + Acetone	B3LYP/6-31+G(d) + $CHCl_3$	X-ray structure
C1-C2	1.401	1.396	1.399	1.399	1.387
O1-C1	1.341	1.348	1.343	1.344	1.358
O1-H1	0.992	0.993	0.991	0.993	0.840
C1-C11	1.428	1.424	1.425	1.423	1.416
C2-H2	1.085	1.085	1.084	1.084	0.950
C3-C4	1.407	1.414	1.411	1.405	1.405
C4-H4	1.083	1.084	1.082	1.085	0.950
C4-C12	1.386	1.385	1.383	1.387	1.377
C5-H5	1.085	1.085	1.085	1.085	0.950
C5-C6	1.410	1.410	1.410	1.411	1.402
C5-C14	1.388	1.388	1.388	1.387	1.385
C6-C7	1.390	1.391	1.390	1.391	1.383
C7-H7	1.086	1.086	1.086	1.086	0.950
C7-C8	1.407	1.406	1.407	1.406	1.397
O8-H8	0.992	0.993	0.992	0.992	0.840
O8-C8	1.342	1.348	1.343	1.346	1.355
O9-C9	1.268	1.272	1.270	1.269	1.266
O10-C10	1.233	1.231	1.230	1.230	1.227
O3-H3	0.972	1.004	0.979	0.973	0.840
O1…O9	2.584	2.580	2.590	2.582	2.568
O8…O9	2.584	2.579	2.582	2.584	2.583
C6-C6′	1.509	1.508	1.509	1.508	1.507

C8-C13	1.416	1.415	1.416	1.416	1.399
C9-C11	1.462	1.450	1.450	1.456	1.450
C9-C13	1.453	1.461	1.464	1.459	1.455
C10-C14	1.491	1.490	1.492	1.489	1.480
C11-C12	1.420	1.421	1.420	1.418	1.404
C13-C14	1.421	1.420	1.419	1.421	1.413
Angle (°)					
O1-H1…O9	146.366	147.161	146.587	146.625	145.57
O8-H8…O9	146.023	146.819	146.356	146.237	145.25
O1-C1-C11	122.281	121.869	122.043	122.223	122.17
O8-C8-C13	122.834	122.460	122.783	122.592	122.69
H3-O3-C3	109.937	111.967	110.845	110.649	109.55
O3-C3-C4	121.199	121.970	121.684	121.793	122.13
O3-C3-C2	117.784	117.977	118.034	117.189	117.16
O1-C1-C2	117.231	117.473	117.432	117.333	117.01
C5-C6-C6′	120.088	120.142	120.112	120.073	120.65
C7-C8-O8	117.260	117.354	117.281	117.297	117.10
Torsion angle					
H1-O1-C1-C11	0.056	-0.078	0.034	-0.051	-0.38
H1-O1-C1-C2	-179.923	-179.890	-179.969	-179.994	-179.63
H8-O8-C8-C13	0.008	-0.090	0.007	-0.037	-0.56
C7-C8-O8-H8	179.968	179.899	-179.996	-179.980	-179.26
H3-O3-C3-C4	-0.024	1.218	0.001	-1.107	0.52
C2-C3-O3-H3	179.978	-178.850	179.994	178.934	179.73
O9-C9-C13-C14	179.942	-179.780	-179.987	-179.955	-178.66
O9-C9-C11-C1	0.007	-0.115	-0.059	-0.033	-1.00
C8-C13-C14-C10	-179.938	-179.946	-179.952	-179.995	179.150
C4-C12-C10-O10	-0.159	0.471	-0.046	0.047	-1.88
C13-C14-C10-O10	179.827	179.685	179.926	-179.937	178.32
O10-C10-C14-C5	0.140	-0.333	-0.047	0.111	0.74
C5-C14-C13-C8	0.027	-0.036	0.022	0.044	0.46
C1-C11-C12-C4	-0.047	-0.198	0.012	-0.088	0.76
O9-C9-C13-C8	-0.065	0.215	0.026	-0.063	0.94

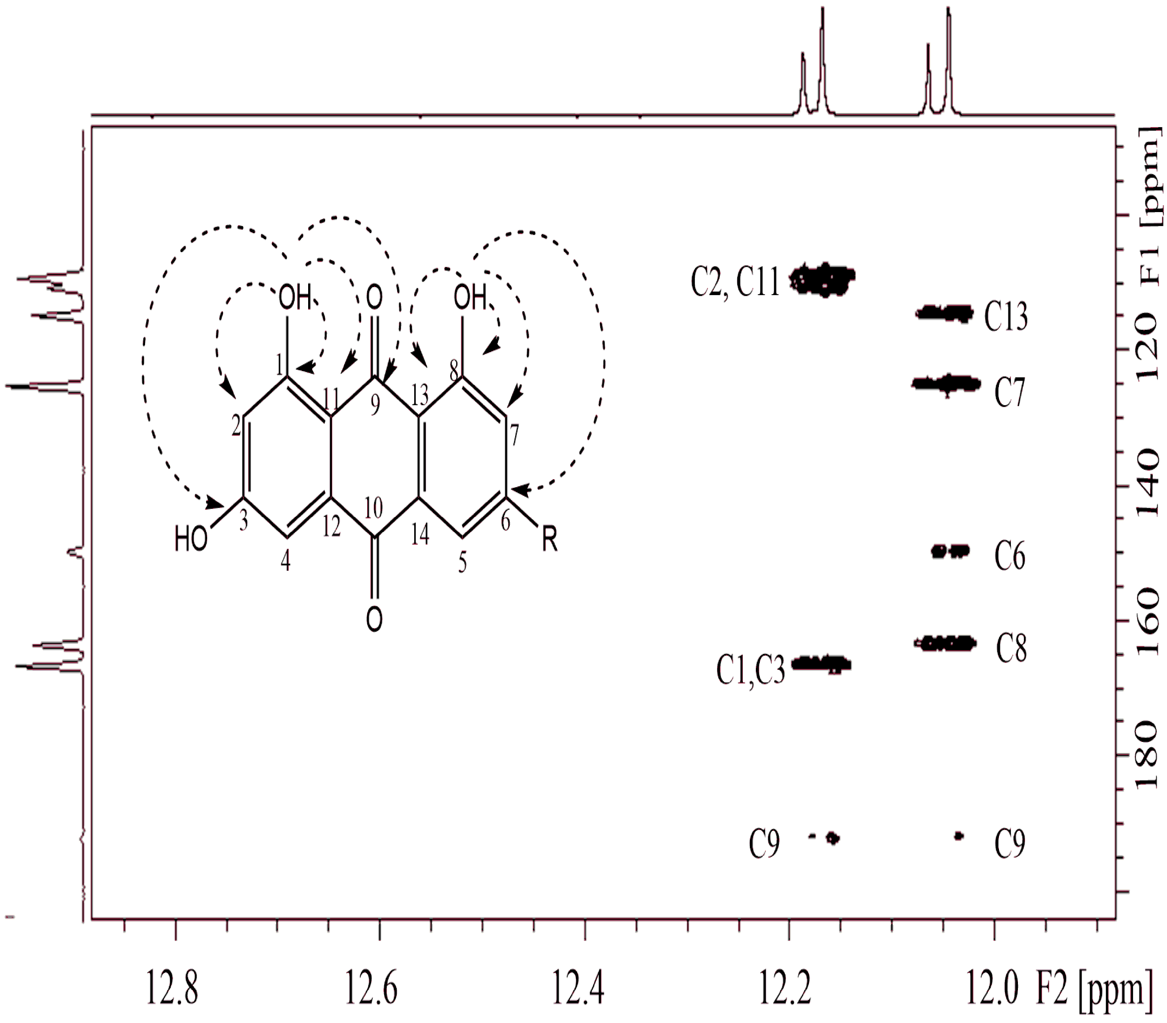

Figure S1. Selected region of the 400 MHz ^{1}H-^{13}C HMBC NMR spectrum of 1 mg of emodin **(2)** in 0.6 ml of acetone-d_6 illustrating critical connectivities for the assignment of the C(1)–OH and C(8)–OH groups. Number of scans 128, number of increments 256, total experimental time 18 hours. The splitting of the –OH resonances in acetone-d_6 is attributed to deuterium isotopic effect (see text).

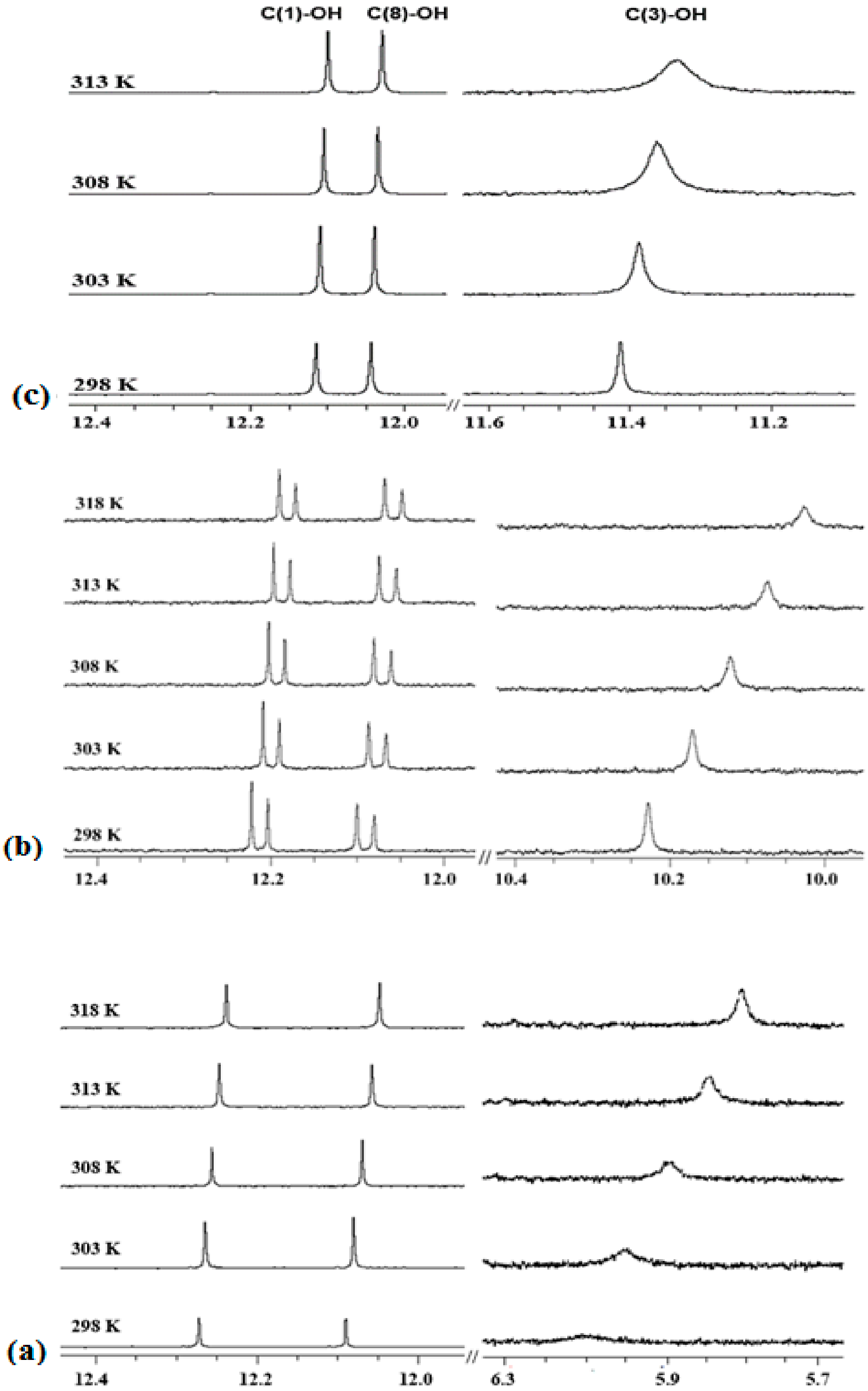

Figure S2. ^{1}H-NMR spectra (500 MHz) of the –OH protons of emodin (**2**), at various temperatures in (**a**) $CDCl_3$, (**b**) acetone-d_6, and (**c**) DMSO-d_6. **(b)** and **(c)** were recorded after the addition of 2 μL of TFA.

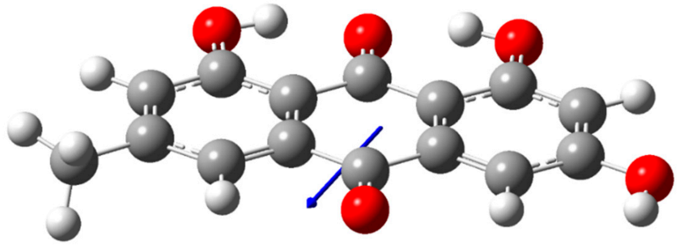

Conformer   A

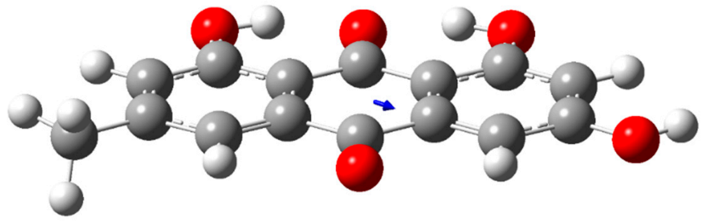

Conformer B

Figure S3. Optimized structures, along with their dipole moments, of the two conformers A and B of emodin (**2**).

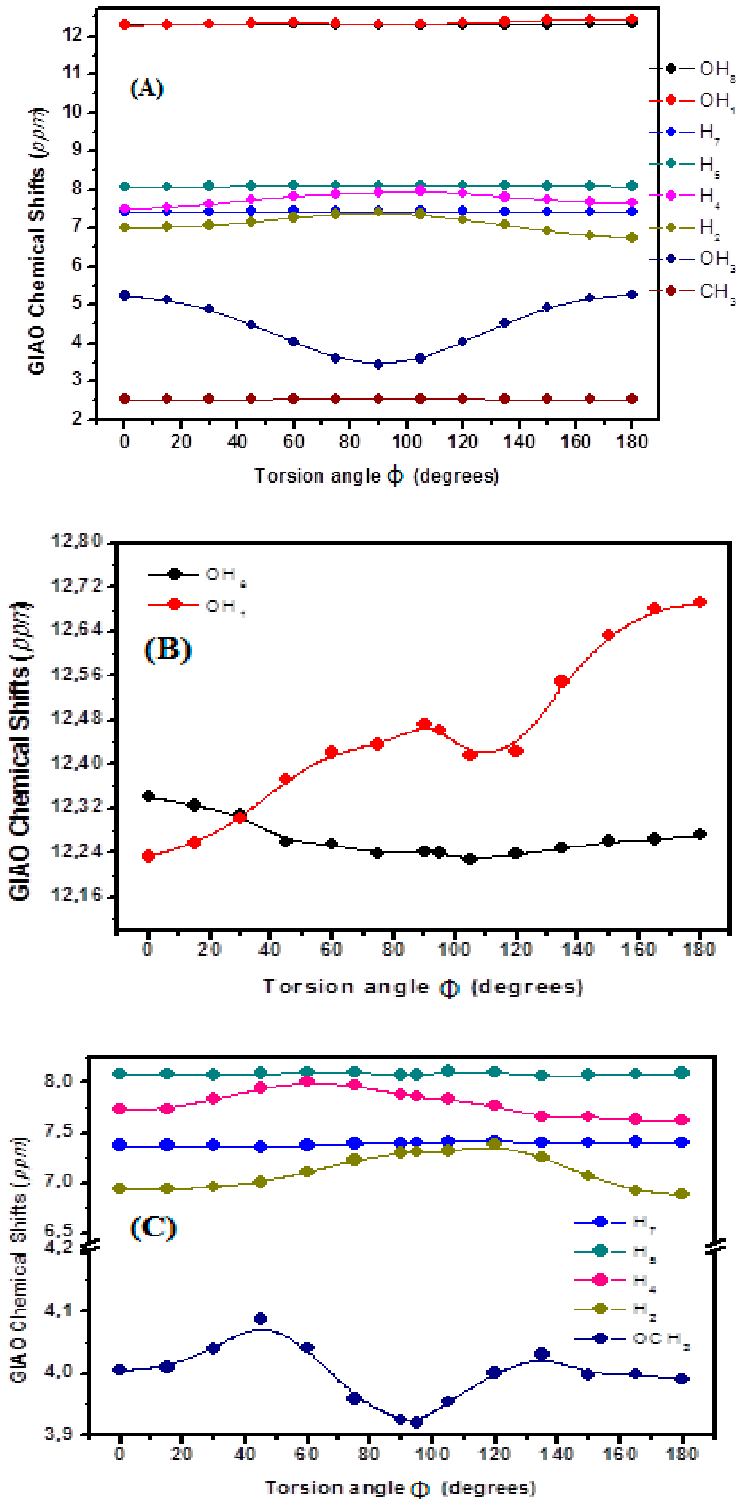

Figure S4. The dependence of calculated, δ_{calc}, GIAO ^{1}H-NMR chemical shifts of physcion **(3)** as a function of the torsion angle φ = C(4)–C(3)–O(3)–C(3) in steps of 15°.

(b)

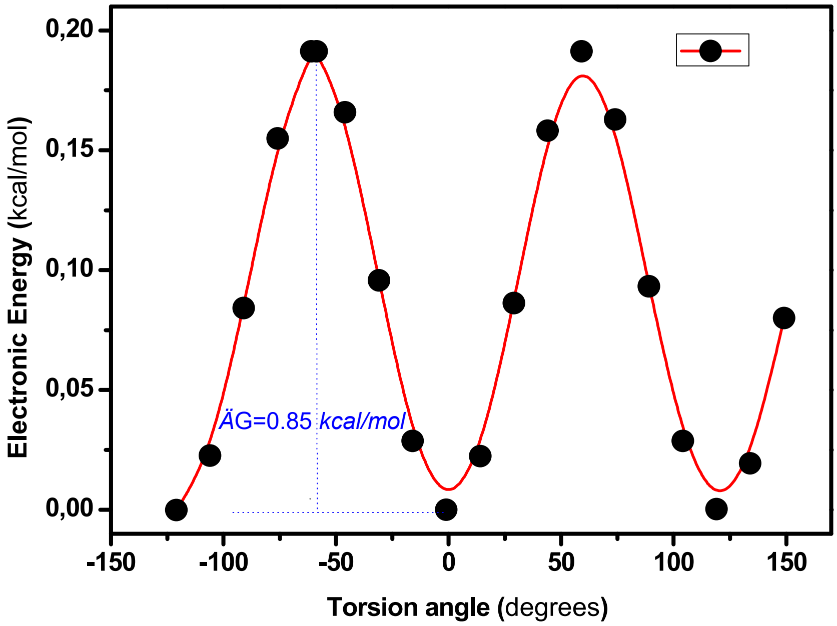

(a)

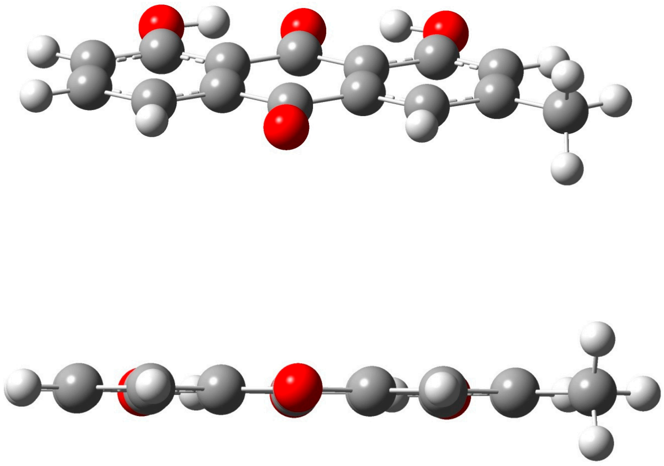

Figure S5. (**a**) Different perspectives of the low energy conformation of the CH_3– group of chyrosophanol (**1**), at the B3LYP/6-31+G(d) (CPCM-$CHCl_3$) level. (**b**) The electronic energy (Hartree unit) of chyrosophanol as a function of the torsion angle C(5)–C(6)–C(6')–H(6').

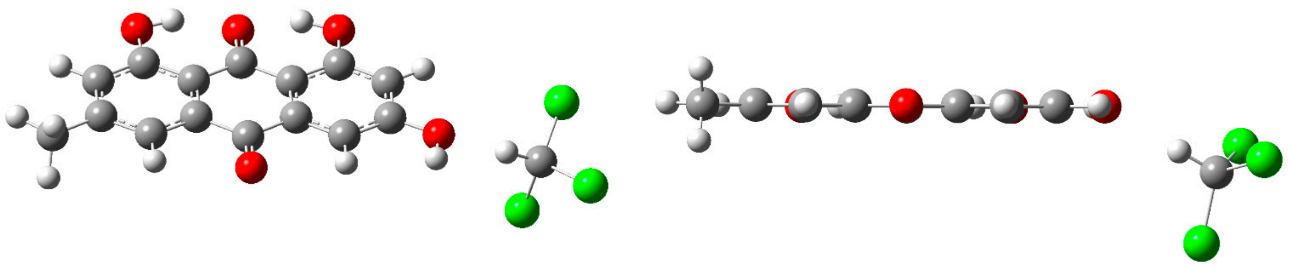

Conformer A

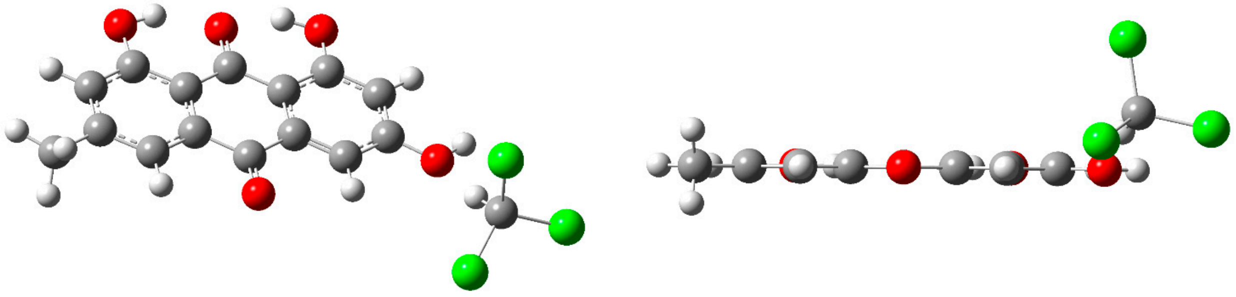

Conformer B

Figure S6. Different perspectives of the B3LYP/6-31+G(d) (CPCM-$CHCl_3$) optimized structures of emodin (**2**) with a single molecule of $CHCl_3$.

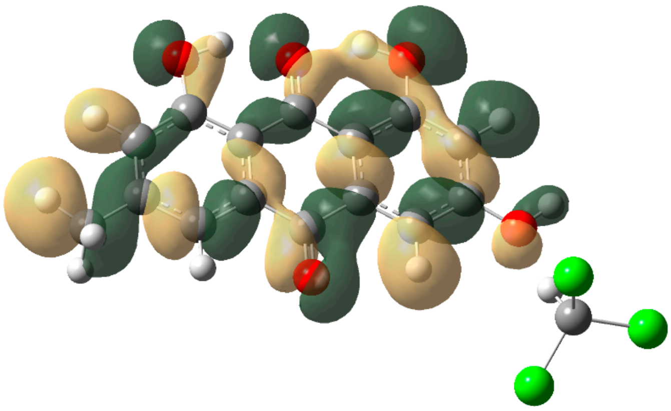

Conformer B

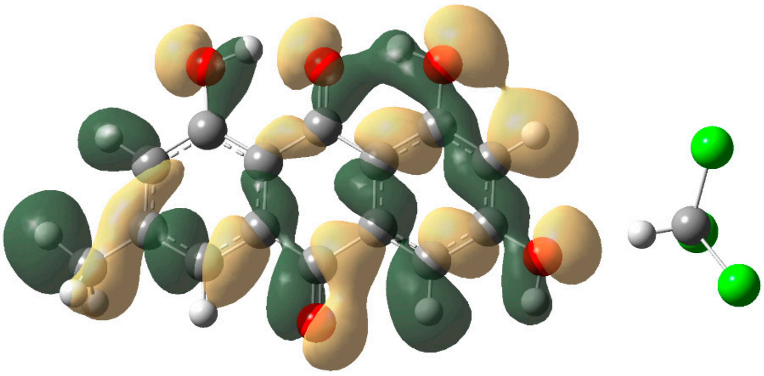

Conformer A

Figure S7. Molecular orbitals for conformers A and B of emodin (**2**) with a single molecule of $CHCl_3$ at the B3LYP/6-31+G(d) (CPCM-$CHCl_3$) level.

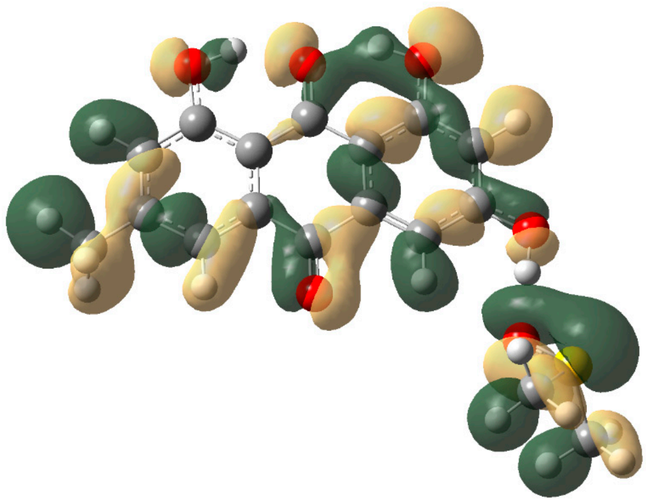

Conformer A2

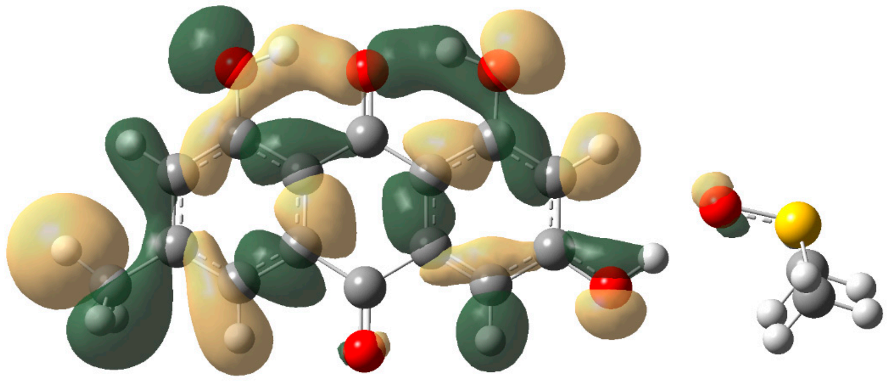

Conformer B

Figure S8. Molecular orbitals for conformers A_2 and B of emodin (**2**) with a single molecule of DMSO at the B3LYP/6-31+G(d) (CPCM-DMSO) level.

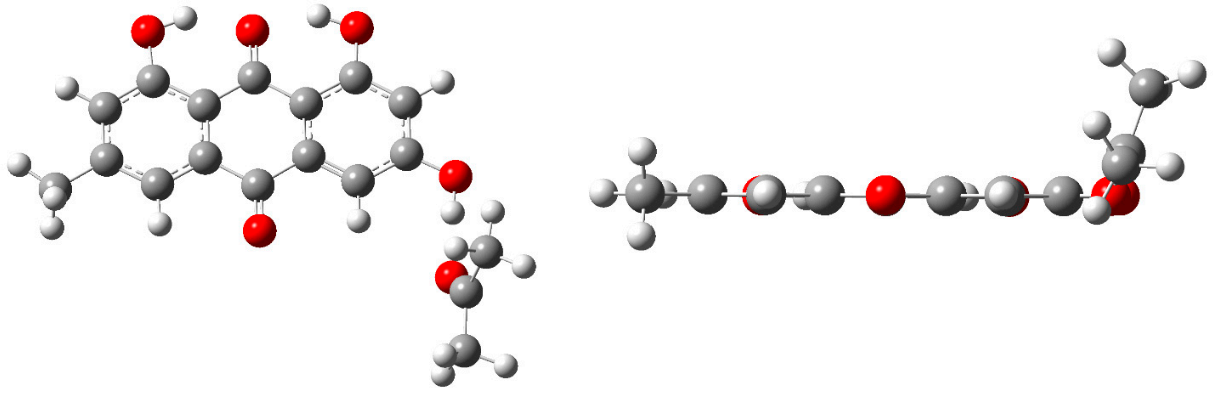

Conformer A

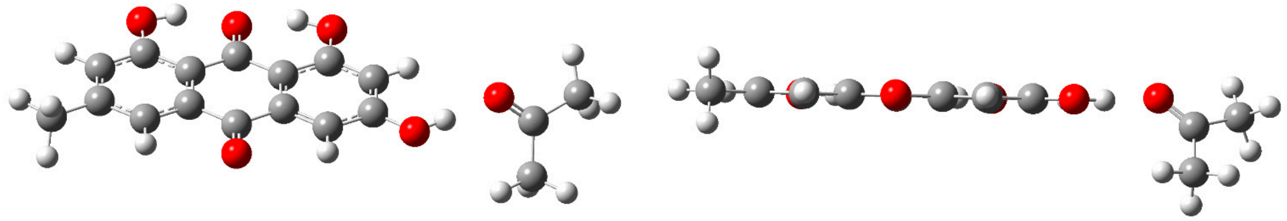

Conformer B:

Figure S9. Different perspectives of the B3LYP/6-31+G(d) (CPCM-acetone) optimized structures of conformers A and B of emodin (**2**) with a discrete molecule of acetone.

(A)

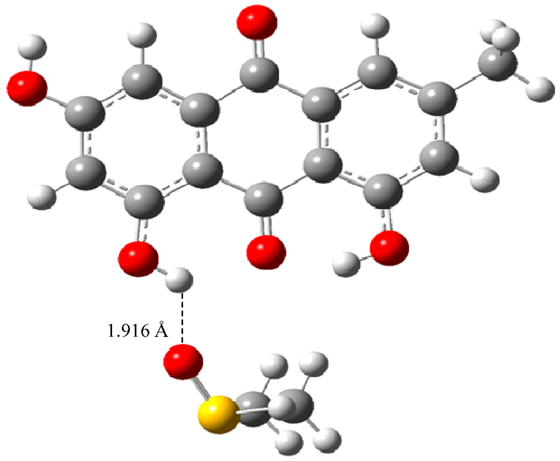

(B)

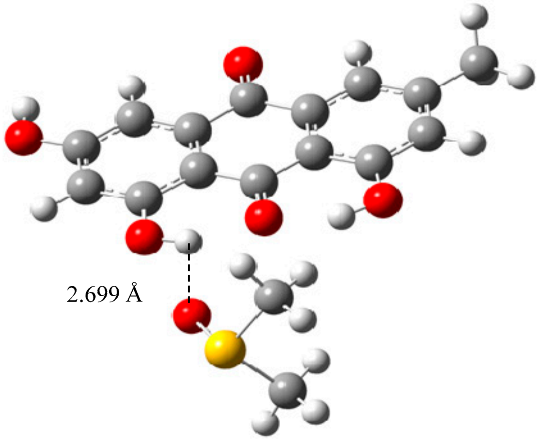

Figure S10. (A) Emodin (**2**) input geometry at the 6-31+G(d) level with a discrete molecule of DMSO at the position of the C1-OH group. **(B)** Emodin minimized structure of (**A**).

Cartesian coordinates of DFT calculated structures

chrysophanol

chrysophanol APFD/6-31+G(d)

Input orientation:

Center Number	Atomic Number	Atomic Type	Coordinates (Angstroms) X	Y	Z
1	6	0	-1.992983	1.454939	-0.019148
2	8	0	-1.892283	2.786028	-0.036849
3	1	0	-0.926018	3.003884	-0.038967
4	6	0	-3.284700	0.904075	-0.012475
5	1	0	-4.126626	1.591859	-0.022256
6	6	0	-3.481412	-0.469296	0.005994
7	6	0	-2.361134	-1.322196	0.018394
8	1	0	-2.483651	-2.402044	0.033121
9	6	0	2.533703	-2.006870	0.029933
10	1	0	2.360051	-3.078460	0.044454
11	6	0	3.828767	-1.473064	0.022933
12	1	0	4.686732	-2.141398	0.032205
13	6	0	4.033341	-0.103461	0.004363
14	1	0	5.031926	0.324614	-0.001156
15	6	0	2.937789	0.776085	-0.007816
16	8	0	3.198629	2.085496	-0.025376
17	1	0	2.326184	2.554696	-0.031896
18	6	0	0.475943	1.150353	-0.013679
19	8	0	0.650736	2.400279	-0.030300
20	6	0	0.074396	-1.740200	0.025647
21	8	0	-0.093219	-2.951464	0.042496
22	6	0	1.441454	-1.152595	0.018016
23	6	0	1.621963	0.250674	-0.001110
24	6	0	-0.866213	0.597670	-0.006953
25	6	0	-1.080074	-0.801645	0.012024
26	6	0	-4.864369	-1.054896	0.012121
27	1	0	-5.016863	-1.684054	0.897328
28	1	0	-5.020241	-1.693600	-0.865722
29	1	0	-5.635423	-0.278939	0.009167

chrysophanol B3LYP/6-31+G(d)

Input orientation:

Center Number	Atomic Number	Atomic Type	Coordinates (Angstroms) X	Y	Z

1	6	0	3.494558	-0.251027	0.000025
2	6	0	2.377666	-1.100179	0.000067
3	6	0	0.909873	0.851453	0.000013
4	6	0	2.051661	1.695463	-0.000041
5	6	0	3.339377	1.127554	-0.000032
6	6	0	-0.436496	1.425689	0.000016
7	6	0	-1.596108	0.541554	0.000046
8	6	0	-1.439692	-0.870807	0.000067
9	6	0	-2.546000	-1.707170	0.000059
10	1	0	-2.383064	-2.780665	0.000063
11	6	0	-3.853778	-1.176654	0.000038
12	6	0	-4.018616	0.204185	0.000020
13	6	0	-2.911680	1.072509	0.000037
14	1	0	2.488118	-2.179055	0.000103
15	1	0	4.193362	1.797893	-0.000073
16	8	0	-0.591635	2.683167	-0.000134
17	6	0	1.097887	-0.556364	0.000060
18	1	0	-5.008788	0.651140	0.000009
19	8	0	1.967704	3.035657	-0.000112
20	1	0	1.005219	3.272944	-0.000127
21	8	0	-3.163789	2.391247	0.000089
22	1	0	-2.290892	2.860769	0.000202
23	6	0	-0.075939	-1.480057	0.000087
24	8	0	0.076291	-2.698335	0.000079
25	6	0	-5.044574	-2.103643	0.000116
26	1	0	-5.031817	-2.756425	-0.881521
27	1	0	-5.032583	-2.755234	0.882654
28	1	0	-5.987756	-1.549193	-0.000648
29	1	0	4.494470	-0.676675	0.000029

----

chrysophanol ωB97XD/6-31+G(d)

Input orientation:

Center Number	Atomic Number	Atomic Type	Coordinates (Angstroms) X	Y	Z
1	6	0	3.493645	-0.245538	-0.008334
2	6	0	2.378920	-1.092931	-0.012612
3	6	0	0.919659	0.855096	0.001642
4	6	0	2.054757	1.695678	0.006017
5	6	0	3.338909	1.127176	0.000970
6	6	0	-0.428368	1.429269	0.006253
7	6	0	-1.588525	0.543084	0.002233
8	6	0	-1.430441	-0.863161	-0.005594
9	6	0	-2.526873	-1.701397	-0.009863
10	1	0	-2.363201	-2.774849	-0.017079
11	6	0	-3.831688	-1.172968	-0.006380
12	6	0	-4.000040	0.201049	-0.000952

13	6	0	-2.897580	1.070433	0.004628
14	1	0	2.489502	-2.171641	-0.020033
15	1	0	4.192266	1.797544	0.004372
16	8	0	-0.583077	2.674007	0.013698
17	6	0	1.107563	-0.546886	-0.007605
18	1	0	-4.990584	0.646299	-0.000467
19	8	0	1.978516	3.029812	0.014843
20	1	0	1.026561	3.277329	0.016878
21	8	0	-3.157490	2.380899	0.010940
22	1	0	-2.297453	2.857699	0.013903
23	6	0	-0.066356	-1.470353	-0.012095
24	8	0	0.085375	-2.680045	-0.020730
25	6	0	-5.015595	-2.103114	0.004027
26	1	0	-4.926199	-2.856490	-0.785637
27	1	0	-5.073295	-2.637079	0.959483
28	1	0	-5.955479	-1.563483	-0.139687
29	1	0	4.492555	-0.671600	-0.012435

----

chrysophanol TPSSh/TZVP

Input orientation:

Center Number	Atomic Number	Atomic Type	Coordinates (Angstroms) X	Y	Z
1	6	0	3.492344	-0.247460	-0.029202
2	6	0	2.382754	-1.098665	-0.018713
3	6	0	0.914490	0.841652	0.003815
4	6	0	2.049224	1.690048	-0.013419
5	6	0	3.334893	1.126579	-0.025832
6	6	0	-0.425902	1.410903	0.000210
7	6	0	-1.579220	0.530790	0.018139
8	6	0	-1.423727	-0.876376	0.005624
9	6	0	-2.528375	-1.708533	0.008888
10	1	0	-2.365118	-2.780129	0.006338
11	6	0	-3.830716	-1.175446	0.011654
12	6	0	-3.995229	0.201477	0.014866
13	6	0	-2.890192	1.065938	0.013773
14	1	0	2.494439	-2.175531	-0.021016
15	1	0	4.186556	1.796049	-0.035130
16	8	0	-0.582870	2.669377	-0.005746
17	6	0	1.104637	-0.560906	-0.008462
18	1	0	-4.984117	0.645882	0.015595
19	8	0	1.949343	3.025410	-0.014240
20	1	0	0.976665	3.234442	-0.010178
21	8	0	-3.126389	2.383847	0.013091
22	1	0	-2.236014	2.827796	0.007042
23	6	0	-0.063709	-1.485466	0.013530
24	8	0	0.088925	-2.700379	0.031026

25	6	0	-5.021263	-2.096815	0.012004
26	1	0	-5.009058	-2.742941	-0.870570
27	1	0	-5.004120	-2.748598	0.890312
28	1	0	-5.958026	-1.538180	0.016417
29	1	0	4.491829	-0.667832	-0.039411

----

chrysophanol M062X_def2TZVP

Input orientation:

Center Number	Atomic Number	Atomic Type	Coordinates (Angstroms) X	Y	Z
1	6	0	3.478963	-0.247443	-0.000061
2	6	0	2.368252	-1.090355	0.000027
3	6	0	0.916906	0.852411	-0.000006
4	6	0	2.048056	1.690419	-0.000088
5	6	0	3.325881	1.121189	-0.000121
6	6	0	-0.430863	1.429649	0.000025
7	6	0	-1.589752	0.541141	0.000035
8	6	0	-1.432002	-0.858382	0.000083
9	6	0	-2.522237	-1.696193	0.000103
10	1	0	-2.355106	-2.765735	0.000136
11	6	0	-3.821902	-1.170119	0.000058
12	6	0	-3.990988	0.199270	0.000002
13	6	0	-2.894072	1.067560	-0.000005
14	1	0	2.471275	-2.166278	0.000065
15	1	0	4.176401	1.789517	-0.000177
16	8	0	-0.583445	2.664051	-0.000312
17	6	0	1.102914	-0.542465	0.000045
18	1	0	-4.979362	0.641404	-0.000025
19	8	0	1.970599	3.021515	-0.000090
20	1	0	1.022873	3.268045	0.000025
21	8	0	-3.151640	2.375319	-0.000005
22	1	0	-2.295077	2.849653	0.000073
23	6	0	-0.069112	-1.466801	0.000080
24	8	0	0.081130	-2.665672	0.000224
25	6	0	-5.001364	-2.098231	0.000084
26	1	0	-4.977129	-2.744790	-0.878642
27	1	0	-4.977301	-2.744511	0.879022
28	1	0	-5.941214	-1.549763	-0.000091
29	1	0	4.475300	-0.670565	-0.000082

Emodin

Emodin_ APFD/6-31+G(d)_ conformation A

Input orientation:

Center Number	Atomic Number	Atomic Type	Coordinates (Angstroms) X	Y	Z
1	6	0	3.486604	-0.246091	0.000134
2	6	0	2.367263	-1.097347	-0.000034
3	6	0	0.906543	0.852043	-0.000214
4	6	0	2.052159	1.694195	-0.000035
5	6	0	3.332495	1.134294	0.000126
6	6	0	-0.425696	1.421904	-0.000378
7	6	0	-1.581367	0.538274	-0.000226
8	6	0	-1.425788	-0.868146	-0.000240
9	6	0	-2.527858	-1.704580	-0.000067
10	1	0	-2.366059	-2.779335	-0.000099
11	6	0	-3.830771	-1.171687	0.000088
12	6	0	-3.995884	0.205910	0.000080
13	6	0	-2.890833	1.073401	-0.000055
14	1	0	2.469487	-2.180408	-0.000061
15	1	0	4.201095	1.785059	0.000272
16	8	0	-0.585264	2.675504	-0.000540
17	6	0	1.098937	-0.547460	-0.000222
18	1	0	-4.987297	0.652114	0.000214
19	8	0	1.961902	3.025172	0.000062
20	1	0	0.996429	3.250646	0.000061
21	8	0	-3.139077	2.385392	0.000071
22	1	0	-2.260928	2.845142	0.000116
23	6	0	-0.068797	-1.473757	-0.000546
24	8	0	0.090715	-2.686345	0.000200
25	6	0	-5.014703	-2.096166	0.000295
26	1	0	-4.999610	-2.748734	-0.881025
27	1	0	-4.999807	-2.748056	0.882124
28	1	0	-5.960545	-1.546624	-0.000012
29	8	0	4.753588	-0.720983	0.000301
30	1	0	4.743575	-1.688787	0.000284

Emodin_ APFD/6-31+G(d)_ conformation B

Input orientation:

Center Number	Atomic Number	Atomic Type	Coordinates (Angstroms) X	Y	Z
1	6	0	3.487767	-0.220819	0.000029

2	6	0	2.374599	-1.077626	0.000040
3	6	0	0.902982	0.866882	0.000005
4	6	0	2.040477	1.715094	-0.000017
5	6	0	3.325488	1.160913	0.000002
6	6	0	-0.430546	1.432036	0.000010
7	6	0	-1.582211	0.544478	0.000016
8	6	0	-1.420432	-0.861324	0.000044
9	6	0	-2.519474	-1.701651	0.000057
10	1	0	-2.353323	-2.775740	0.000079
11	6	0	-3.824364	-1.173869	0.000053
12	6	0	-3.995419	0.203170	0.000023
13	6	0	-2.893972	1.074599	0.000000
14	1	0	2.508652	-2.154457	0.000064
15	1	0	4.177123	1.837812	-0.000017
16	8	0	-0.594223	2.686064	0.000120
17	6	0	1.105495	-0.534077	0.000032
18	1	0	-4.988692	0.645216	0.000002
19	8	0	1.946094	3.045766	-0.000086
20	1	0	0.977641	3.264545	-0.000161
21	8	0	-3.146707	2.386045	-0.000071
22	1	0	-2.269994	2.848187	-0.000150
23	6	0	-0.060777	-1.463995	0.000076
24	8	0	0.099041	-2.675524	0.000041
25	6	0	-5.004571	-2.103085	0.000111
26	1	0	-4.986609	-2.755610	-0.881187
27	1	0	-4.986975	-2.755008	0.881866
28	1	0	-5.952680	-1.557415	-0.000263
29	8	0	4.702356	-0.815433	0.000041
30	1	0	5.402366	-0.146877	0.000029

Emodin_ B3LYP/6-31+G(d)_conformation A

Input orientation:

Center Number	Atomic Number	Atomic Type	Coordinates (Angstroms) X	Y	Z
1	6	0	3.500404	-0.245954	0.000035
2	6	0	2.379553	-1.095699	0.000009
3	6	0	0.914306	0.858908	-0.000050
4	6	0	2.066381	1.697837	-0.000030
5	6	0	3.349381	1.137052	0.000016
6	6	0	-0.424939	1.429825	-0.000080
7	6	0	-1.586412	0.542667	-0.000038
8	6	0	-1.429134	-0.868972	-0.000017
9	6	0	-2.534447	-1.707882	0.000035
10	1	0	-2.370446	-2.781201	0.000045
11	6	0	-3.842533	-1.179077	0.000071

12	6	0	-4.007832	0.201592	0.000043
13	6	0	-2.901403	1.071563	-0.000012
14	1	0	2.485110	-2.177223	0.000022
15	1	0	4.217421	1.787218	0.000031
16	8	0	-0.585764	2.688108	0.000009
17	6	0	1.106506	-0.546027	-0.000032
18	1	0	-4.998368	0.647751	0.000057
19	8	0	1.978657	3.036418	-0.000060
20	1	0	1.014712	3.270856	-0.000119
21	8	0	-3.155212	2.390181	-0.000051
22	1	0	-2.281293	2.859151	-0.000122
23	6	0	-0.066163	-1.475204	-0.000057
24	8	0	0.094845	-2.692436	0.000041
25	6	0	-5.032907	-2.107050	0.000166
26	1	0	-5.020607	-2.759469	-0.881755
27	1	0	-5.020922	-2.758862	0.882545
28	1	0	-5.976268	-1.552906	-0.000184
29	8	0	4.770948	-0.733005	0.000076
30	1	0	4.756933	-1.703614	0.000082

Emodin_ B3LYP/6-31+G(d)_conformation B

Input orientation:

Center Number	Atomic Number	Atomic Type	Coordinates (Angstroms) X	Y	Z
1	6	0	3.501404	-0.222147	0.000134
2	6	0	2.385745	-1.079157	-0.000036
3	6	0	0.910544	0.872155	-0.000196
4	6	0	2.054528	1.716898	-0.000028
5	6	0	3.341642	1.160473	0.000131
6	6	0	-0.429785	1.438570	-0.000318
7	6	0	-1.587557	0.548118	-0.000209
8	6	0	-1.424605	-0.862932	-0.000229
9	6	0	-2.527163	-1.705350	-0.000059
10	1	0	-2.359192	-2.778049	-0.000089
11	6	0	-3.836989	-1.181172	0.000098
12	6	0	-4.007700	0.199021	0.000084
13	6	0	-2.904602	1.072541	-0.000059
14	1	0	2.520298	-2.154783	-0.000060
15	1	0	4.194450	1.834502	0.000268
16	8	0	-0.594180	2.697395	-0.000308
17	6	0	1.112573	-0.534430	-0.000217
18	1	0	-4.999940	0.641377	0.000204
19	8	0	1.963168	3.055242	0.000043
20	1	0	0.996473	3.284037	-0.000014
21	8	0	-3.162421	2.390750	0.000004
22	1	0	-2.289936	2.862211	-0.000047

23	6	0	-0.059208	-1.466758	-0.000520
24	8	0	0.101464	-2.682959	0.000147
25	6	0	-5.024034	-2.113403	0.000297
26	1	0	-5.009337	-2.765789	-0.881600
27	1	0	-5.009456	-2.765241	0.882604
28	1	0	-5.969424	-1.562687	0.000071
29	8	0	4.725342	-0.815499	0.000292
30	1	0	5.427011	-0.144637	0.000399

Emodin_ ωB97XD/6-31+G(d)_conformation A

Input orientation:

Center Number	Atomic Number	Atomic Type	Coordinates (Angstroms) X	Y	Z
1	6	0	3.487908	-0.246818	0.123628
2	6	0	2.367480	-1.093430	0.131149
3	6	0	0.913736	0.858107	0.043510
4	6	0	2.060736	1.692161	0.038046
5	6	0	3.338992	1.130140	0.078000
6	6	0	-0.425336	1.430330	-0.000512
7	6	0	-1.588620	0.542766	0.001925
8	6	0	-1.432202	-0.862375	0.048242
9	6	0	-2.528809	-1.701663	0.051146
10	1	0	-2.365958	-2.774525	0.090194
11	6	0	-3.832650	-1.173869	0.006503
12	6	0	-3.998998	0.199760	-0.036357
13	6	0	-2.896126	1.069292	-0.040294
14	1	0	2.469517	-2.174546	0.168281
15	1	0	4.207579	1.778962	0.072840
16	8	0	-0.583252	2.675465	-0.041010
17	6	0	1.103893	-0.540121	0.091233
18	1	0	-4.988981	0.645225	-0.068910
19	8	0	1.981805	3.023689	-0.004738
20	1	0	1.028337	3.267663	-0.028416
21	8	0	-3.155675	2.379347	-0.083542
22	1	0	-2.294515	2.855173	-0.079938
23	6	0	-0.070258	-1.467497	0.099680
24	8	0	0.088290	-2.675503	0.146169
25	6	0	-5.017545	-2.103025	-0.008546
26	1	0	-5.075105	-2.632873	-0.966393
27	1	0	-4.930931	-2.859469	0.778331
28	1	0	-5.956907	-1.562474	0.135390
29	8	0	4.749015	-0.733001	0.160463
30	1	0	4.734096	-1.698125	0.188177

Emodin_ωB97XD /6-31+G(d)_conformation B

Input orientation:

Center Number	Atomic Number	Atomic Type	Coordinates (Angstroms) X	Y	Z
1	6	0	3.490569	-0.224039	0.018513
2	6	0	2.375373	-1.077811	0.028621
3	6	0	0.910679	0.871216	-0.005108
4	6	0	2.049287	1.710780	-0.014672
5	6	0	3.332691	1.153169	-0.002955
6	6	0	-0.429946	1.439326	-0.017546
7	6	0	-1.589393	0.548156	-0.009301
8	6	0	-1.426647	-0.856993	0.011290
9	6	0	-2.520334	-1.699924	0.019584
10	1	0	-2.352902	-2.772671	0.037192
11	6	0	-3.826527	-1.176454	0.007136
12	6	0	-3.998916	0.197108	-0.010619
13	6	0	-2.899507	1.070438	-0.020099
14	1	0	2.508987	-2.153381	0.046017
15	1	0	4.184310	1.827985	-0.010729
16	8	0	-0.591874	2.685448	-0.035683
17	6	0	1.111183	-0.529740	0.016810
18	1	0	-4.990958	0.639086	-0.018124
19	8	0	1.967166	3.042655	-0.034932
20	1	0	1.011158	3.282026	-0.040338
21	8	0	-3.163898	2.380420	-0.038136
22	1	0	-2.304239	2.858758	-0.042290
23	6	0	-0.061640	-1.460336	0.027669
24	8	0	0.097138	-2.668108	0.048883
25	6	0	-5.007909	-2.110120	-0.000292
26	1	0	-5.068023	-2.642671	-0.956420
27	1	0	-4.914135	-2.864412	0.787957
28	1	0	-5.948661	-1.572991	0.147423
29	8	0	4.705556	-0.815661	0.030825
30	1	0	5.405027	-0.149819	0.021962

Emodin_ TPSSh/TZVP_conformation A

Input orientation:

Center Number	Atomic Number	Atomic Type	Coordinates (Angstroms) X	Y	Z
1	6	0	3.488213	-0.249399	-0.035446
2	6	0	2.372745	-1.100394	-0.020363
3	6	0	0.910173	0.844778	0.017813

4	6	0	2.055910	1.686673	-0.009696
5	6	0	3.336320	1.130058	-0.023809
6	6	0	-0.422065	1.412243	0.002940
7	6	0	-1.578361	0.530131	0.017751
8	6	0	-1.423962	-0.876561	0.008470
9	6	0	-2.528738	-1.710054	0.010882
10	1	0	-2.365515	-2.781663	0.010487
11	6	0	-3.830660	-1.177300	0.011119
12	6	0	-3.993588	0.199795	0.012091
13	6	0	-2.887988	1.064537	0.011944
14	1	0	2.478088	-2.180345	-0.028708
15	1	0	4.202035	1.779237	-0.038383
16	8	0	-0.583148	2.671668	-0.004782
17	6	0	1.102258	-0.555005	-0.011216
18	1	0	-4.982155	0.644988	0.010808
19	8	0	1.952501	3.020584	-0.010632
20	1	0	0.978056	3.226203	-0.006784
21	8	0	-3.124725	2.382748	0.009395
22	1	0	-2.233335	2.825860	0.003600
23	6	0	-0.065552	-1.483938	0.019581
24	8	0	0.095063	-2.697884	0.042525
25	6	0	-5.022061	-2.097940	0.010647
26	1	0	-5.009371	-2.745495	-0.870864
27	1	0	-5.007908	-2.748109	0.890205
28	1	0	-5.958132	-1.538094	0.012264
29	8	0	4.758572	-0.733515	-0.055759
30	1	0	4.734614	-1.700333	-0.059487

Emodin_ TPSSh/TZVP_conformation B

Input orientation:

Center Number	Atomic Number	Atomic Type	Coordinates (Angstroms) X	Y	Z
1	6	0	3.489805	-0.226830	-0.036262
2	6	0	2.379650	-1.084910	-0.021998
3	6	0	0.907129	0.857376	0.015488
4	6	0	2.044678	1.705532	-0.010803
5	6	0	3.328811	1.152552	-0.026734
6	6	0	-0.426094	1.420530	0.002687
7	6	0	-1.579106	0.535714	0.017334
8	6	0	-1.419194	-0.870472	0.007587
9	6	0	-2.521217	-1.707418	0.010308
10	1	0	-2.354184	-2.778429	0.009532
11	6	0	-3.824844	-1.179009	0.011248
12	6	0	-3.993019	0.197589	0.012767
13	6	0	-2.890740	1.066035	0.012428
14	1	0	2.512319	-2.158798	-0.030059

15	1	0	4.180335	1.824768	-0.040039
16	8	0	-0.590215	2.680794	-0.003987
17	6	0	1.109229	-0.543973	-0.009996
18	1	0	-4.983313	0.638991	0.012157
19	8	0	1.937426	3.038936	-0.011549
20	1	0	0.959924	3.238402	-0.006969
21	8	0	-3.131532	2.383751	0.010709
22	1	0	-2.241458	2.829421	0.005130
23	6	0	-0.058319	-1.475786	0.017875
24	8	0	0.101592	-2.688705	0.040827
25	6	0	-5.013081	-2.103764	0.010966
26	1	0	-4.998690	-2.750594	-0.871055
27	1	0	-4.995892	-2.754608	0.889967
28	1	0	-5.951173	-1.547252	0.013714
29	8	0	4.714520	-0.815535	-0.055160
30	1	0	5.403258	-0.136456	-0.061646

Emodin_M062X_def2TZVP _conformation A

Input orientation:

Center Number	Atomic Number	Atomic Type	Coordinates (Angstroms) X	Y	Z
1	6	0	3.475146	-0.248450	0.000122
2	6	0	2.358961	-1.091164	-0.000022
3	6	0	0.911622	0.855115	-0.000178
4	6	0	2.054017	1.687791	-0.000036
5	6	0	3.326893	1.125480	0.000106
6	6	0	-0.427187	1.430427	-0.000270
7	6	0	-1.588989	0.540421	-0.000190
8	6	0	-1.432639	-0.858741	-0.000205
9	6	0	-2.522836	-1.697565	-0.000044
10	1	0	-2.355932	-2.767165	-0.000074
11	6	0	-3.822183	-1.171493	0.000096
12	6	0	-3.989664	0.197975	0.000077
13	6	0	-2.892189	1.066536	-0.000059
14	1	0	2.460725	-2.169533	-0.000048
15	1	0	4.194146	1.770537	0.000230
16	8	0	-0.584205	2.666189	-0.000476
17	6	0	1.100513	-0.537030	-0.000190
18	1	0	-4.977772	0.640863	0.000187
19	8	0	1.971611	3.016684	0.000040
20	1	0	1.021410	3.258631	0.000036
21	8	0	-3.149611	2.374513	0.000016
22	1	0	-2.291413	2.847426	0.000007
23	6	0	-0.071094	-1.465872	-0.000508
24	8	0	0.086690	-2.663821	0.000248
25	6	0	-5.002425	-2.098926	0.000278

26	1	0	-4.979365	-2.745478	-0.878492
27	1	0	-4.979413	-2.745051	0.879366
28	1	0	-5.941700	-1.549416	0.000126
29	8	0	4.733105	-0.735881	0.000271
30	1	0	4.714287	-1.698459	0.000261

Emodin_ M062X_def2TZVP_conformation B

Input orientation:

Center Number	Atomic Number	Atomic Type	Coordinates (Angstroms) X	Y	Z
1	6	0	3.476822	-0.227221	-0.000217
2	6	0	2.365287	-1.076909	-0.000068
3	6	0	0.908582	0.866855	-0.000026
4	6	0	2.042993	1.704866	-0.000163
5	6	0	3.319828	1.146281	-0.000269
6	6	0	-0.430905	1.438330	0.000067
7	6	0	-1.589538	0.545625	0.000062
8	6	0	-1.428012	-0.853073	0.000101
9	6	0	-2.515877	-1.694895	0.000120
10	1	0	-2.345568	-2.763945	0.000146
11	6	0	-3.816770	-1.172935	0.000064
12	6	0	-3.989138	0.196106	0.000005
13	6	0	-2.894641	1.067791	0.000013
14	1	0	2.494944	-2.149708	-0.000044
15	1	0	4.172119	1.814734	-0.000352
16	8	0	-0.590482	2.674790	-0.000581
17	6	0	1.106899	-0.527324	0.000008
18	1	0	-4.978758	0.635551	-0.000019
19	8	0	1.957855	3.033512	-0.000090
20	1	0	1.004843	3.269673	0.000189
21	8	0	-3.155715	2.375317	0.000066
22	1	0	-2.298685	2.850166	0.000236
23	6	0	-0.064291	-1.458363	0.000052
24	8	0	0.092715	-2.655528	0.000275
25	6	0	-4.994026	-2.104147	0.000083
26	1	0	-4.968707	-2.750616	-0.878675
27	1	0	-4.968912	-2.750314	0.879070
28	1	0	-5.935106	-1.557713	-0.000117
29	8	0	4.691852	-0.812324	-0.000292
30	1	0	5.384390	-0.143514	-0.000454

EMODIN COMPLEXES

Emodin_CHCl3_B3LYP/6-31+G(d)_conformation A (cpcm =chloroform)

Input orientation:

Center Number	Atomic Number	Atomic Type	Coordinates (Angstroms) X	Y	Z
1	8	0	6.431330	1.076948	3.783352
2	8	0	6.218106	6.266593	5.347314
3	8	0	4.288727	0.556519	5.127922
4	1	0	5.074082	0.400920	4.540220
5	8	0	2.212856	4.028740	7.510312
6	1	0	2.288648	4.971865	7.734473
7	8	0	8.579629	1.545474	2.424139
8	1	0	7.822585	1.030380	2.806700
9	6	0	6.381586	2.291351	4.149409
10	6	0	5.308461	2.752657	5.018981
11	6	0	5.239517	4.106499	5.435624
12	6	0	6.272728	5.090725	4.988860
13	6	0	7.363068	4.598354	4.100746
14	6	0	7.401104	3.235187	3.700992
15	6	0	4.300844	1.856091	5.473268
16	6	0	3.273729	2.308435	6.309787
17	1	0	2.515664	1.610997	6.649986
18	6	0	3.236869	3.644163	6.697208
19	6	0	4.218522	4.551936	6.262531
20	1	0	4.192364	5.595232	6.561753
21	6	0	8.457585	2.814962	2.856170
22	6	0	9.433583	3.736288	2.435213
23	1	0	10.227687	3.374020	1.788360
24	6	0	9.388261	5.068810	2.832119
25	6	0	8.336665	5.490567	3.673888
26	1	0	8.277452	6.524210	3.999717
27	6	0	10.434321	6.055549	2.377209
28	1	0	10.925892	6.526650	3.236920
29	1	0	9.978434	6.860121	1.787005
30	1	0	11.201782	5.574875	1.763915
31	6	0	-0.936770	3.020086	7.426950
32	1	0	0.105940	3.325363	7.427204
33	17	0	-0.999246	1.274203	7.819037
34	17	0	-1.789859	3.989587	8.668812
35	17	0	-1.602420	3.337295	5.794827

Emodin_CHCl3_B3LYP/6-31+G(d)_conformation B (cpcm =chloroform)

Input orientation:

Center Number	Atomic Number	Atomic Type	Coordinates (Angstroms) X	Y	Z
1	8	0	7.400169	3.464292	3.819357
2	8	0	3.693626	-0.224480	5.266343
3	8	0	6.144087	5.286023	5.146372
4	1	0	6.841884	4.872163	4.572224
5	8	0	2.170097	4.005334	7.424430
6	1	0	2.178167	4.941438	7.687372
7	8	0	8.697733	1.675019	2.480216
8	1	0	8.478120	2.563991	2.862840
9	6	0	6.533122	2.600774	4.159327
10	6	0	5.410313	2.981125	5.003663
11	6	0	4.431068	2.028365	5.391025
12	6	0	4.537673	0.607317	4.936620
13	6	0	5.697239	0.238178	4.075921
14	6	0	6.657104	1.218788	3.706101
15	6	0	5.266161	4.318329	5.463221
16	6	0	4.182148	4.676082	6.276664
17	1	0	4.098889	5.705928	6.612535
18	6	0	3.242086	3.715247	6.635691
19	6	0	3.360630	2.385079	6.195191
20	1	0	2.623532	1.644480	6.483510
21	6	0	7.739930	0.819083	2.884831
22	6	0	7.846669	-0.516912	2.458605
23	1	0	8.691873	-0.783103	1.830114
24	6	0	6.903048	-1.470342	2.827235
25	6	0	5.821827	-1.074834	3.643790
26	1	0	5.070634	-1.798110	3.944943
27	6	0	7.021448	-2.903249	2.371479
28	1	0	6.131902	-3.207059	1.806399
29	1	0	7.102026	-3.579153	3.231706
30	1	0	7.898975	-3.052389	1.736025
31	6	0	-0.943886	2.907047	7.493177
32	1	0	0.113097	3.151446	7.436742
33	17	0	-1.091925	1.187176	7.965028
34	17	0	-1.683108	3.978509	8.725335
35	17	0	-1.660493	3.196351	5.877587

Emodin_DMSO_B3LYP/6-31+G(d)_conformation A(cpcm =DMSO)

Input orientation:

Center Number	Atomic Number	Atomic Type	Coordinates (Angstroms) X	Y	Z
1	8	0	7.181064	2.820011	4.992641
2	8	0	4.132198	5.731398	1.573311
3	8	0	5.071483	1.696507	5.962252
4	1	0	6.032594	1.912755	5.832140
5	8	0	0.873881	2.906509	4.242159
6	1	0	0.400984	3.492271	3.576703
7	8	0	9.320963	3.916439	4.059939
8	1	0	8.748872	3.329003	4.621087
9	6	0	6.461376	3.501533	4.194393
10	6	0	5.018616	3.355814	4.196375
11	6	0	4.203636	4.110417	3.308728
12	6	0	4.825721	5.073005	2.350051
13	6	0	6.310309	5.210363	2.364652
14	6	0	7.091154	4.439539	3.266936
15	6	0	4.374992	2.451629	5.088780
16	6	0	2.984619	2.317708	5.086384
17	1	0	2.512720	1.623494	5.774123
18	6	0	2.206462	3.072049	4.204703
19	6	0	2.824697	3.974947	3.308393
20	1	0	2.223349	4.560791	2.622091
21	6	0	8.497132	4.603945	3.242737
22	6	0	9.087467	5.509713	2.343882
23	1	0	10.169562	5.606325	2.356043
24	6	0	8.314948	6.261272	1.463411
25	6	0	6.913398	6.099427	1.484801
26	1	0	6.287385	6.672465	0.808247
27	6	0	8.951138	7.233805	0.501905
28	1	0	8.593491	8.254046	0.687521
29	1	0	8.689805	6.985330	-0.533876
30	1	0	10.041140	7.231823	0.590906
31	16	0	-1.867117	4.733152	2.310237
32	8	0	-0.348640	4.446380	2.456373
33	6	0	-2.695975	3.110223	2.345469
34	1	0	-3.777597	3.271058	2.330709
35	1	0	-2.388857	2.574194	1.444709
36	1	0	-2.395055	2.561343	3.241612
37	6	0	-2.424884	5.357363	3.929617
38	1	0	-1.927525	6.316345	4.090336
39	1	0	-3.508093	5.503517	3.891755
40	1	0	-2.153993	4.647021	4.715076

Emodin_DMSO_B3LYP/6-31+G(d)_conformation B(cpcm =DMSO)

Input orientation:

Center Number	Atomic Number	Atomic Type	Coordinates (Angstroms) X	Y	Z
1	8	0	7.117201	4.015855	3.810138
2	8	0	4.422726	0.607503	7.066177
3	8	0	4.906011	4.747594	2.701115
4	1	0	5.881046	4.702036	2.888224
5	8	0	0.888849	2.753982	4.270835
6	1	0	0.462678	3.354261	3.586684
7	8	0	9.351085	3.332057	4.900214
8	1	0	8.720610	3.801264	4.292092
9	6	0	6.479431	3.222473	4.574195
10	6	0	5.037274	3.110342	4.481809
11	6	0	4.313658	2.223698	5.327904
12	6	0	5.034656	1.385309	6.332337
13	6	0	6.516848	1.521985	6.417999
14	6	0	7.204528	2.417741	5.556254
15	6	0	4.300969	3.885302	3.542509
16	6	0	2.908654	3.777699	3.459504
17	1	0	2.372138	4.381710	2.734704
18	6	0	2.223808	2.899594	4.304428
19	6	0	2.936639	2.118303	5.242464
20	1	0	2.398972	1.438899	5.894120
21	6	0	8.612287	2.511467	5.675043
22	6	0	9.294857	1.733497	6.626623
23	1	0	10.374921	1.834195	6.686772
24	6	0	8.613077	0.859702	7.468318
25	6	0	7.210489	0.762236	7.350867
26	1	0	6.654902	0.087612	7.994435
27	6	0	9.347250	0.030959	8.492715
28	1	0	9.142316	-1.036878	8.350381
29	1	0	9.019356	0.288643	9.507400
30	1	0	10.428523	0.184494	8.433910
31	16	0	-1.634953	4.821157	2.255096
32	8	0	-0.167609	4.345828	2.427961
33	6	0	-2.652795	3.311301	2.176252
34	1	0	-3.705802	3.603674	2.134104
35	1	0	-2.376833	2.787504	1.258334
36	1	0	-2.456761	2.685057	3.050500
37	6	0	-2.171502	5.425031	3.889132
38	1	0	-1.578118	6.314460	4.111950
39	1	0	-3.230753	5.690929	3.829676
40	1	0	-2.004332	4.651930	4.643866

Emodin_Acetone_B3LYP/6-31+G(d)_conformation A(cpcm =acetone)

Input orientation:

Center Number	Atomic Number	Atomic Type	Coordinates (Angstroms) X	Y	Z
1	8	0	7.174928	2.886959	5.145715
2	8	0	4.159663	5.672636	1.595638
3	8	0	5.077932	1.651387	6.000294
4	1	0	6.032229	1.916890	5.922281
5	8	0	0.918163	2.655845	4.072745
6	1	0	0.453520	3.218496	3.398158
7	8	0	9.303502	4.092752	4.324963
8	1	0	8.735106	3.474747	4.855720
9	6	0	6.464516	3.537593	4.315145
10	6	0	5.031401	3.320351	4.243447
11	6	0	4.226299	4.040476	3.320111
12	6	0	4.846540	5.041302	2.399848
13	6	0	6.320256	5.249084	2.487561
14	6	0	7.092403	4.511090	3.424281
15	6	0	4.390628	2.377061	5.096138
16	6	0	3.010500	2.172275	5.019969
17	1	0	2.539446	1.448945	5.677486
18	6	0	2.243662	2.893891	4.103583
19	6	0	2.857420	3.835616	3.247646
20	1	0	2.266561	4.398757	2.533739
21	6	0	8.488242	4.743783	3.470699
22	6	0	9.077096	5.683021	2.605673
23	1	0	10.151179	5.832414	2.672288
24	6	0	8.313201	6.401351	1.690757
25	6	0	6.921709	6.171941	1.642303
26	1	0	6.302300	6.718404	0.938257
27	6	0	8.947961	7.407543	0.763588
28	1	0	8.510955	8.402663	0.910899
29	1	0	8.776362	7.133290	-0.284506
30	1	0	10.026990	7.479300	0.926612
31	8	0	-0.388747	4.210588	2.251214
32	6	0	-1.582787	4.178373	1.945662
33	6	0	-2.147606	5.161324	0.953714
34	1	0	-2.763086	4.646770	0.207080
35	1	0	-2.808680	5.860946	1.482299
36	1	0	-1.347862	5.721188	0.464552
37	6	0	-2.528035	3.165810	2.539891
38	1	0	-3.450026	3.652280	2.877678
39	1	0	-2.815772	2.449235	1.759216
40	1	0	-2.064375	2.625799	3.368071

Emodin_Acetone_B3LYP/6-31+G(d)_conformation B(cpcm =acetone)

Input orientation:

Center Number	Atomic Number	Atomic Type	Coordinates (Angstroms) X	Y	Z
1	8	0	7.114710	4.022400	3.925414
2	8	0	4.413419	0.472154	7.018978
3	8	0	4.918179	4.729555	2.771789
4	1	0	5.888199	4.704845	2.986502
5	8	0	0.909714	2.590663	4.169947
6	1	0	0.492995	3.176282	3.483519
7	8	0	9.336021	3.354131	5.052344
8	1	0	8.711723	3.828512	4.442249
9	6	0	6.476833	3.192965	4.649062
10	6	0	5.039823	3.049641	4.512954
11	6	0	4.315109	2.125047	5.315412
12	6	0	5.027765	1.278688	6.319510
13	6	0	6.504170	1.443123	6.445938
14	6	0	7.193855	2.376787	5.626926
15	6	0	4.312649	3.830422	3.572095
16	6	0	2.926031	3.689021	3.444398
17	1	0	2.399229	4.298923	2.717475
18	6	0	2.241999	2.772755	4.246130
19	6	0	2.944034	1.987510	5.186770
20	1	0	2.405126	1.278772	5.805193
21	6	0	8.596072	2.496190	5.783781
22	6	0	9.271461	1.704367	6.729144
23	1	0	10.347392	1.825135	6.818957
24	6	0	8.587821	0.792620	7.527837
25	6	0	7.190565	0.671084	7.373821
26	1	0	6.633619	-0.032761	7.984046
27	6	0	9.314206	-0.052934	8.543996
28	1	0	9.143924	-1.119980	8.356569
29	1	0	8.949053	0.157644	9.556765
30	1	0	10.392028	0.131674	8.524002
31	8	0	-0.225891	4.205785	2.284522
32	6	0	-1.399409	4.241078	1.907881
33	6	0	-1.836559	5.221467	0.851078
34	1	0	-2.420484	4.714813	0.074087
35	1	0	-2.498274	5.969476	1.307382
36	1	0	-0.974189	5.723916	0.408248
37	6	0	-2.443985	3.315377	2.476529
38	1	0	-3.341528	3.877541	2.758631
39	1	0	-2.748339	2.602030	1.699414
40	1	0	-2.061066	2.765334	3.338662

Physcion

Physcion_APFD/6-31+G(d)_ conformation A

Input orientation:

Center Number	Atomic Number	Atomic Type	Coordinates (Angstroms) X	Y	Z
1	6	0	3.482551	-0.263706	0.000098
2	6	0	2.358936	-1.111298	-0.000106
3	6	0	0.899820	0.845397	-0.000182
4	6	0	2.048046	1.685309	-0.000014
5	6	0	3.322917	1.120711	0.000136
6	6	0	-0.431665	1.417518	-0.000229
7	6	0	-1.588746	0.535503	-0.000153
8	6	0	-1.434215	-0.870647	-0.000242
9	6	0	-2.537166	-1.705956	-0.000113
10	1	0	-2.376402	-2.780864	-0.000197
11	6	0	-3.839502	-1.171703	0.000097
12	6	0	-4.003482	0.206058	0.000157
13	6	0	-2.897487	1.072242	0.000039
14	1	0	2.440022	-2.192354	-0.000170
15	1	0	4.194131	1.768189	0.000268
16	8	0	-0.589980	2.671181	0.000077
17	6	0	1.091949	-0.552570	-0.000251
18	1	0	-4.994509	0.653165	0.000302
19	8	0	1.960843	3.018048	-0.000029
20	1	0	0.996569	3.245106	-0.000257
21	8	0	-3.143650	2.384769	0.000131
22	1	0	-2.264103	2.842144	0.000025
23	6	0	-0.077018	-1.477281	-0.000529
24	8	0	0.078783	-2.690570	-0.000072
25	6	0	-5.024407	-2.095022	0.000323
26	1	0	-5.009865	-2.747962	-0.880754
27	1	0	-5.010516	-2.746632	0.882404
28	1	0	-5.969704	-1.544480	-0.000419
29	8	0	4.757575	-0.700765	0.000244
30	6	0	4.998222	-2.098047	0.000185
31	1	0	6.083127	-2.207809	0.000310
32	1	0	4.582024	-2.572241	0.897097
33	1	0	4.582242	-2.572127	-0.896889

Physcion_APFD/6-31+G(d)_ conformation B

Input orientation:

Center Number	Atomic Number	Atomic Type	Coordinates (Angstroms) X	Y	Z
1	6	0	3.435080	-0.274453	-0.119252
2	6	0	2.711338	-0.051586	1.069545
3	6	0	0.791071	0.671552	-0.243740
4	6	0	1.532578	0.440641	-1.428713
5	6	0	2.850732	-0.031041	-1.359700
6	6	0	-0.568310	1.161479	-0.313848
7	6	0	-1.307069	1.394995	0.917229
8	6	0	-0.718351	1.147589	2.180210
9	6	0	-1.425369	1.372500	3.348296
10	1	0	-0.938117	1.170213	4.298540
11	6	0	-2.746988	1.853740	3.303775
12	6	0	-3.334647	2.099831	2.070841
13	6	0	-2.636119	1.877985	0.872946
14	1	0	3.173781	-0.243707	2.032380
15	1	0	3.374586	-0.190982	-2.295439
16	8	0	-1.112134	1.386713	-1.433943
17	6	0	1.415718	0.412550	1.001995
18	1	0	-4.353893	2.471468	1.999391
19	8	0	1.027621	0.655240	-2.645149
20	1	0	0.099181	0.982836	-2.515743
21	8	0	-3.275339	2.139803	-0.270399
22	1	0	-2.644930	1.930232	-1.006261
23	6	0	0.676755	0.640112	2.276766
24	8	0	1.199808	0.418335	3.358947
25	6	0	-3.498150	2.092853	4.582547
26	1	0	-3.578297	1.167975	5.166532
27	1	0	-2.975418	2.824281	5.210680
28	1	0	-4.509582	2.465965	4.396635
29	8	0	4.692180	-0.725574	0.056092
30	6	0	5.490444	-0.970024	-1.088561
31	1	0	6.450750	-1.320289	-0.709100
32	1	0	5.042571	-1.743652	-1.724291
33	1	0	5.638080	-0.051570	-1.669769

Physcion_B3LYP/6-31+G(d)_ conformation A

Input orientation:

Center Number	Atomic Number	Atomic Type	Coordinates (Angstroms) X	Y	Z
1	6	0	3.643233	-1.289311	-0.001223

2	6	0	2.411958	-1.973057	-0.001778
3	6	0	1.230431	0.171062	-0.001011
4	6	0	2.488818	0.841640	-0.000403
5	6	0	3.676891	0.106656	-0.000538
6	6	0	-0.016761	0.922063	-0.000919
7	6	0	-1.290071	0.204313	-0.001435
8	6	0	-1.328616	-1.215136	-0.002027
9	6	0	-2.539111	-1.893560	-0.002417
10	1	0	-2.524653	-2.979240	-0.002881
11	6	0	-3.761691	-1.189376	-0.002222
12	6	0	-3.735183	0.200947	-0.001701
13	6	0	-2.519270	0.909784	-0.001321
14	1	0	2.346919	-3.053614	-0.002311
15	1	0	4.627639	0.628676	-0.000066
16	8	0	-0.002524	2.190470	-0.000382
17	6	0	1.228557	-1.245458	-0.001668
18	1	0	-4.654751	0.779437	-0.001608
19	8	0	2.588443	2.181125	0.000386
20	1	0	1.667153	2.547062	0.000598
21	8	0	-2.588001	2.251091	-0.000982
22	1	0	-1.657152	2.593917	-0.000945
23	6	0	-0.061434	-2.003391	-0.002281
24	8	0	-0.072618	-3.231422	-0.002940
25	6	0	-5.068750	-1.944375	-0.002531
26	1	0	-5.146283	-2.592416	-0.884384
27	1	0	-5.147214	-2.591447	0.879963
28	1	0	-5.926672	-1.265358	-0.003330
29	8	0	4.849224	-1.907543	-0.001217
30	6	0	4.899283	-3.334834	-0.001870
31	1	0	5.960339	-3.587215	-0.001822
32	1	0	4.422342	-3.747048	0.895140
33	1	0	4.422618	-3.746201	-0.899414

Physcion_B3LYP/6-31+G(d)_ conformation B

Input orientation:

Center Number	Atomic Number	Atomic Type	Coordinates (Angstroms) X	Y	Z
1	6	0	1.449524	10.915758	2.934834
2	1	0	0.599262	10.939024	3.609699
3	6	0	1.944682	9.690701	2.441278
4	6	0	3.035618	9.706452	1.578643
5	1	0	3.446323	8.784348	1.176881
6	6	0	3.647601	10.913774	1.194865
7	6	0	3.151123	12.146316	1.688838
8	6	0	3.821969	15.902931	1.459951

9	6	0	3.303410	17.107804	1.962729
10	1	0	3.784918	18.027530	1.654139
11	6	0	2.208948	17.084145	2.824957
12	6	0	1.616972	15.857741	3.198948
13	1	0	0.766071	15.849659	3.870663
14	6	0	2.125116	14.671408	2.704566
15	6	0	1.474138	13.388713	3.118477
16	6	0	2.036613	12.118942	2.569119
17	6	0	3.772770	13.410669	1.300906
18	6	0	3.240542	14.658957	1.821157
19	6	0	1.293841	8.391799	2.850768
20	1	0	1.776966	7.531889	2.377130
21	1	0	0.232196	8.381423	2.574623
22	1	0	1.344662	8.254523	3.938125
23	6	0	2.166816	19.472169	3.029086
24	1	0	3.208498	19.565174	3.359461
25	1	0	1.546351	20.191041	3.565356
26	1	0	2.099949	19.658830	1.950442
27	8	0	4.696840	10.834792	0.359542
28	1	0	5.008908	11.761067	0.190336
29	8	0	4.876890	15.991672	0.635127
30	1	0	5.121048	15.065771	0.370255
31	8	0	1.637279	18.189085	3.359720
32	8	0	0.512838	13.383407	3.881035
33	8	0	4.770340	13.415059	0.514690

Physcion_ωB97XD/6-31+G(d)_ conformation A

Input orientation:

Center Number	Atomic Number	Atomic Type	Coordinates (Angstroms) X	Y	Z
1	6	0	3.486130	-0.263878	-0.008833
2	6	0	2.361471	-1.107047	-0.012662
3	6	0	0.907940	0.852730	0.005830
4	6	0	2.057425	1.684980	0.008916
5	6	0	3.330402	1.118011	0.001519
6	6	0	-0.430853	1.427746	0.014674
7	6	0	-1.595574	0.542036	0.011380
8	6	0	-1.439709	-0.863540	-0.001320
9	6	0	-2.537132	-1.701733	-0.003719
10	1	0	-2.374891	-2.775401	-0.012583
11	6	0	-3.840921	-1.171948	0.006560
12	6	0	-4.006546	0.202348	0.021092
13	6	0	-2.902739	1.070697	0.022354
14	1	0	2.440805	-2.186957	-0.020999
15	1	0	4.201352	1.763849	0.004224
16	8	0	-0.587786	2.673538	0.025062

17	6	0	1.098466	-0.544335	-0.005389
18	1	0	-4.996410	0.649124	0.031710
19	8	0	1.980647	3.018933	0.018922
20	1	0	1.028041	3.264869	0.023740
21	8	0	-3.160789	2.381850	0.035410
22	1	0	-2.298394	2.855733	0.034985
23	6	0	-0.076929	-1.470400	-0.009747
24	8	0	0.078350	-2.679870	-0.019785
25	6	0	-5.026867	-2.099807	-0.010694
26	1	0	-5.090326	-2.621838	-0.972360
27	1	0	-4.936390	-2.862888	0.769514
28	1	0	-5.964905	-1.559813	0.143659
29	8	0	4.756580	-0.711373	-0.014648
30	6	0	4.989087	-2.109765	-0.024166
31	1	0	6.072619	-2.226658	-0.026710
32	1	0	4.571482	-2.585651	0.870337
33	1	0	4.568505	-2.573950	-0.923402

Physcion_ωB97XD/6-31+G(d)_ conformation B

Input orientation:

Center Number	Atomic Number	Atomic Type	Coordinates (Angstroms) X	Y	Z
1	6	0	3.219526	-0.782388	-0.141574
2	6	0	2.500707	-0.533000	1.045027
3	6	0	0.718382	0.493318	-0.260446
4	6	0	1.454519	0.233960	-1.437451
5	6	0	2.701966	-0.403238	-1.371887
6	6	0	-0.576093	1.153767	-0.323348
7	6	0	-1.314105	1.407544	0.913972
8	6	0	-0.787149	1.019494	2.168399
9	6	0	-1.483149	1.257947	3.336845
10	1	0	-1.043650	0.944540	4.279035
11	6	0	-2.737467	1.894857	3.304817
12	6	0	-3.263936	2.279054	2.083418
13	6	0	-2.571515	2.045689	0.884379
14	1	0	2.914218	-0.833573	2.000960
15	1	0	3.221109	-0.576448	-2.306550
16	8	0	-1.059462	1.504848	-1.429585
17	6	0	1.277399	0.091305	0.978733
18	1	0	-4.229179	2.772582	2.017542
19	8	0	1.017775	0.571701	-2.652991
20	1	0	0.139601	1.005294	-2.543959
21	8	0	-3.158412	2.451800	-0.245906
22	1	0	-2.559819	2.221866	-0.992403
23	6	0	0.539986	0.341029	2.256416
24	8	0	1.007456	0.001310	3.329236

25	6	0	-3.480570	2.147325	4.590057
26	1	0	-3.674289	1.205200	5.114559
27	1	0	-2.886809	2.778477	5.260397
28	1	0	-4.438653	2.643081	4.412202
29	8	0	4.405374	-1.396530	0.023501
30	6	0	5.188779	-1.683242	-1.121417
31	1	0	6.088335	-2.171581	-0.747625
32	1	0	4.657946	-2.360015	-1.801129
33	1	0	5.463041	-0.763039	-1.650474

Physcion_ TPSSh/TZVP _ conformation A

Input orientation:

Center Number	Atomic Number	Atomic Type	Coordinates (Angstroms) X	Y	Z
1	6	0	3.484826	-0.253116	0.004238
2	6	0	2.366184	-1.102464	-0.001433
3	6	0	0.901996	0.848293	-0.037535
4	6	0	2.049290	1.689769	-0.018839
5	6	0	3.325097	1.130326	-0.008945
6	6	0	-0.430055	1.415844	-0.018653
7	6	0	-1.586816	0.534198	-0.026167
8	6	0	-1.431512	-0.872041	-0.014091
9	6	0	-2.535934	-1.705994	-0.010121
10	1	0	-2.372315	-2.777531	-0.007194
11	6	0	-3.837974	-1.173527	-0.007322
12	6	0	-4.001627	0.203466	-0.011464
13	6	0	-2.896367	1.068663	-0.017453
14	1	0	2.452304	-2.180325	0.007999
15	1	0	4.192665	1.777385	-0.001374
16	8	0	-0.591584	2.675434	-0.013400
17	6	0	1.096188	-0.549755	-0.006873
18	1	0	-4.990475	0.648127	-0.008042
19	8	0	1.947116	3.025412	-0.022018
20	1	0	0.973666	3.231757	-0.021692
21	8	0	-3.132898	2.386979	-0.017821
22	1	0	-2.240692	2.829356	-0.017065
23	6	0	-0.072007	-1.478609	-0.027201
24	8	0	0.086480	-2.693170	-0.045324
25	6	0	-5.029253	-2.094500	-0.000447
26	1	0	-5.019390	-2.745173	-0.879723
27	1	0	-5.012322	-2.741518	0.881394
28	1	0	-5.965466	-1.534812	0.002088
29	8	0	4.764541	-0.696270	0.018158
30	6	0	4.991629	-2.109399	0.028645
31	1	0	6.073093	-2.224486	0.037122
32	1	0	4.563420	-2.568700	0.924028

33	1	0	4.576266	-2.579710	-0.867063

Physcion_ TPSSh/TZVP _ conformation B

Input orientation:

Center Number	Atomic Number	Atomic Type	Coordinates (Angstroms) X	Y	Z
1	6	0	3.510487	-0.140280	-0.024974
2	6	0	2.407469	-1.015411	-0.012748
3	6	0	0.902123	0.898790	0.017353
4	6	0	2.025490	1.762330	-0.006404
5	6	0	3.323923	1.237078	-0.018990
6	6	0	-0.438663	1.438876	0.003257
7	6	0	-1.576630	0.533881	0.017363
8	6	0	-1.392684	-0.869461	0.008836
9	6	0	-2.480598	-1.725070	0.010495
10	1	0	-2.295144	-2.793042	0.010910
11	6	0	-3.792669	-1.218996	0.008711
12	6	0	-3.984277	0.154736	0.008791
13	6	0	-2.897087	1.041713	0.009520
14	1	0	2.561412	-2.086607	-0.018033
15	1	0	4.149080	1.935622	-0.030509
16	8	0	-0.625609	2.696725	-0.005313
17	6	0	1.129082	-0.500915	-0.003690
18	1	0	-4.982023	0.579070	0.006124
19	8	0	1.896094	3.094254	-0.009133
20	1	0	0.915088	3.277253	-0.006142
21	8	0	-3.159920	2.355489	0.006316
22	1	0	-2.276661	2.815341	0.001836
23	6	0	-0.021612	-1.452548	0.022173
24	8	0	0.157177	-2.663044	0.045783
25	6	0	-4.965590	-2.163293	0.007263
26	1	0	-4.939644	-2.810224	-0.874437
27	1	0	-4.939213	-2.813364	0.886620
28	1	0	-5.912678	-1.622133	0.008438
29	8	0	4.723855	-0.739629	-0.039707
30	6	0	5.886755	0.092677	-0.049354
31	1	0	6.731319	-0.592331	-0.058452
32	1	0	5.909433	0.721562	-0.944332
33	1	0	5.926285	0.718576	0.847119

Physcion M062X_def2TZVP_ conformation A

Input orientation:

Center Number	Atomic Number	Atomic Type	Coordinates (Angstroms) X	Y	Z

1	6	0	3.475325	-0.263585	0.000026
2	6	0	2.355492	-1.103834	-0.000047
3	6	0	0.908593	0.850116	-0.000130
4	6	0	2.053647	1.681398	-0.000061
5	6	0	3.320436	1.114839	0.000013
6	6	0	-0.429928	1.427588	-0.000168
7	6	0	-1.593053	0.539582	-0.000104
8	6	0	-1.437448	-0.859222	-0.000116
9	6	0	-2.528422	-1.697092	-0.000011
10	1	0	-2.362497	-2.766831	-0.000032
11	6	0	-3.827259	-1.169812	0.000091
12	6	0	-3.993830	0.199816	0.000084
13	6	0	-2.895551	1.067211	-0.000011
14	1	0	2.432059	-2.180440	-0.000062
15	1	0	4.190298	1.756612	0.000074
16	8	0	-0.585516	2.663325	-0.000252
17	6	0	1.097815	-0.539717	-0.000135
18	1	0	-4.981602	0.643446	0.000159
19	8	0	1.973741	3.012114	-0.000029
20	1	0	1.024774	3.255711	-0.000037
21	8	0	-3.151001	2.375705	0.000026
22	1	0	-2.291635	2.846684	-0.000010
23	6	0	-0.075528	-1.466948	-0.000303
24	8	0	0.078465	-2.665523	0.000113
25	6	0	-5.008410	-2.096195	0.000219
26	1	0	-4.986019	-2.742747	-0.878565
27	1	0	-4.986109	-2.742372	0.879284
28	1	0	-5.947166	-1.545768	0.000060
29	8	0	4.742200	-0.712346	0.000102
30	6	0	4.959165	-2.110862	0.000124
31	1	0	6.036880	-2.245653	0.000185
32	1	0	4.531979	-2.573478	0.892523
33	1	0	4.532078	-2.573492	-0.892314

Physcion M062X_def2TZVP_ conformation B

Input orientation:

Center Number	Atomic Number	Atomic Type	Coordinates (Angstroms) X	Y	Z
1	6	0	3.475171	-0.150774	-0.024238
2	6	0	2.365947	-1.012417	-0.020421
3	6	0	0.885457	0.910295	-0.012693
4	6	0	2.009153	1.758003	-0.016769
5	6	0	3.299294	1.221685	-0.022522
6	6	0	-0.459247	1.464818	-0.006189
7	6	0	-1.606669	0.556915	-0.001847

8	6	0	-1.427783	-0.839729	-0.004000
9	6	0	-2.505587	-1.694776	0.000030
10	1	0	-2.322260	-2.761648	-0.001822
11	6	0	-3.812513	-1.188892	0.006450
12	6	0	-4.001699	0.178095	0.008684
13	6	0	-2.918128	1.062951	0.004589
14	1	0	2.511944	-2.083251	-0.021826
15	1	0	4.125398	1.916358	-0.025417
16	8	0	-0.635954	2.699591	-0.004212
17	6	0	1.102173	-0.483700	-0.014741
18	1	0	-4.996629	0.605355	0.013733
19	8	0	1.908707	3.085857	-0.015322
20	1	0	0.952929	3.311084	-0.010776
21	8	0	-3.194729	2.367474	0.006997
22	1	0	-2.342667	2.851797	0.003270
23	6	0	-0.056546	-1.429260	-0.010648
24	8	0	0.113502	-2.624830	-0.012415
25	6	0	-4.978858	-2.133828	0.010685
26	1	0	-4.950111	-2.779241	-0.868746
27	1	0	-4.942947	-2.780404	0.889010
28	1	0	-5.926137	-1.598131	0.014932
29	8	0	4.676327	-0.751156	-0.029270
30	6	0	5.831437	0.064912	-0.033691
31	1	0	6.679428	-0.613514	-0.037231
32	1	0	5.860918	0.694403	-0.926117
33	1	0	5.868080	0.693807	0.858870
